# Phosphoinositides: Regulators of Nervous System Function in Health and Disease

**DOI:** 10.3389/fnmol.2019.00208

**Published:** 2019-08-23

**Authors:** Padinjat Raghu, Annu Joseph, Harini Krishnan, Pramod Singh, Sankhanil Saha

**Affiliations:** National Centre for Biological Sciences-TIFR, Bengaluru, India

**Keywords:** phosphoinositides, brain disease, genetics, cellular organelles, inherited disorders, human genetics and genomics, neurological disorders

## Abstract

Phosphoinositides, the seven phosphorylated derivatives of phosphatidylinositol have emerged as regulators of key sub-cellular processes such as membrane transport, cytoskeletal function and plasma membrane signaling in eukaryotic cells. All of these processes are also present in the cells that constitute the nervous system of animals and in this setting too, these are likely to tune key aspects of cell biology in relation to the unique structure and function of neurons. Phosphoinositides metabolism and function are mediated by enzymes and proteins that are conserved in evolution, and analysis of knockouts of these in animal models implicate this signaling system in neural function. Most recently, with the advent of human genome analysis, mutations in genes encoding components of the phosphoinositide signaling pathway have been implicated in human diseases although the cell biological basis of disease phenotypes in many cases remains unclear. In this review we evaluate existing evidence for the involvement of phosphoinositide signaling in human nervous system diseases and discuss ways of enhancing our understanding of the role of this pathway in the human nervous system’s function in health and disease.

## Historical Perspective

Phosphoinositides are low abundance cellular membrane lipids generated by phosphorylation on the inositol headgroup of phosphatidylinositol. Historically, studies on brain tissue played an important part in the discovery of these molecules. Biochemical fractionation studies by Jordi Folch-Pi on brain extracts lead to the discovery of a mixture (which he named diphosphoinositide) that contained mainly what we now know to be phosphatidylinositol (PI), phosphatidylinositol 4 phosphate (PI4P) and phosphatidylinositol 4,5 bisphosphate [PI(4,5)P_2_] (Folch and Wolleey, 1942; Folch, 1949a, b). The signaling functions of phosphoinositides originated from observations by Hokin and Hokin (1955) that stimulation of pancreatic slices led to the incorporation of phosphate in lipids. It was also reported that stimulation of brain tissue led to the incorporation of phosphate into phosphatidic acid and phosphoinositides (Dawson, 1954a, b); the Hokins’ also discovered that stimulation of nervous system tissue (brain slices or dorsal root ganglion) with acetylcholine, resulted in a similar incorporation of phosphate into lipid fractions (Hokin et al., 1960). Subsequently, it became apparent that in cells, the activity of a phosphoinositide phospholipase C (Rhee and Choi, 1992) results in the hydrolysis of phosphoinositides leading to generation the products, soluble inositol 1,4,5 triphosphate (IP_3_) and diacyl glycerol (DAG) that act as second messengers (Berridge and Irvine, 1984). In the subsequent years, numerous studies have demonstrated the ability of phosphoinositides to regulate key cellular functions through non-PLC mediated mechanisms in a range of cell types. These include actin dynamics, vesicular transport and nuclear function which have all been extensively reviewed (Divecha et al., 1993; Martin, 1998; Simonsen et al., 2001; Haucke, 2005; Di Paolo and De Camilli, 2006; Wu et al., 2014; Posor et al., 2015). Although phosphoinositides are present in every cell type in eukaryota, given the long-established observation that inositol lipids are enriched in the brain, these lipids are likely to support key cellular functions in the human brain and alterations in these could lead to diseases of the nervous system. In this review, we provide a review of the major known functions of phosphoinositides in controlling neural cell function and human disease.

## Overview of Phosphoinositide Signaling

**FIGURE 1:**
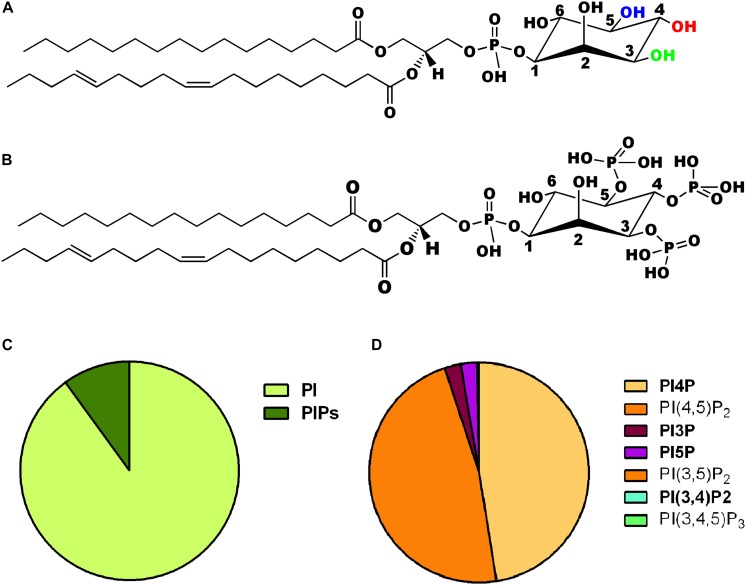
**(A)** Chemical structure of phosphatidylinositol. The three hydroxyl groups at positions 3, 4, and 5 on the inositol ring are shown in green, red, and blue, respectively. **(B)** Chemical structure of one of the seven phosphoinositides, phosphatidylinositol 3,4,5 trisphosphate is shown where the hydroxyls are position 3,4,5 of the inositol headgroup are all phosphorylated. **(C)** Pie chart representing the relative abundance of phosphatidylinositol (PI) and its phosphorylated derivatives (PIPs) in animal cells. **(D)** Pie chart representing the relative abundance of the seven PIPs in animal cells. The two most abundant forms PI4P and PI(4,5)P_2_ are shown individually along with the other five low abundance derivatives [PI3P, PI5P, PI(3,5)P_2_, PI(3,4)P_2_, and PI(3,4,5)P_3_].

**FIGURE 2:**
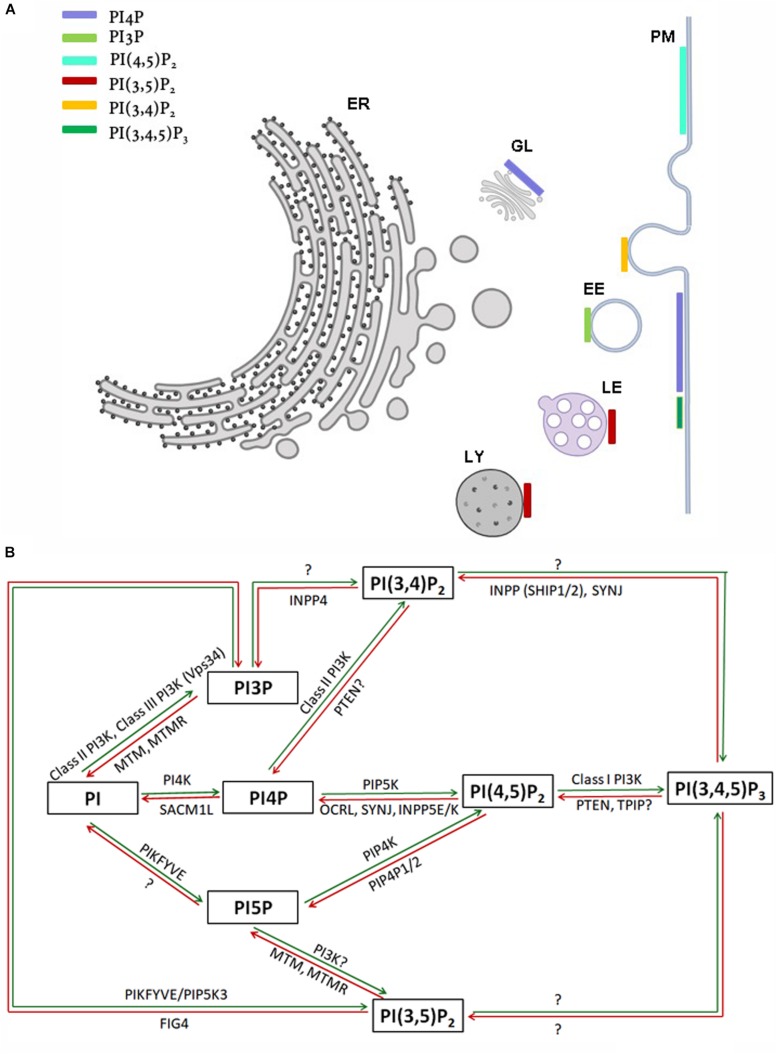
**(A)** Sub-cellular distribution of phosphoinositides in a eukaryotic cell. Major organelles are shown: PM-plasma membrane, ER-endoplasmic reticulum, EE-early endosome, LE-late endosome, LY-lysosome, GL-Golgi. The presence of a major pool of a specific phosphoinositide at a particular organelle membrane is indicated by a colored bar. Minor pools of each species at organelles though reported are not represented. **(B)** Schematic representation of kinases and phosphatases regulating phosphoinositide metabolism. Green and red arrows represent phosphorylation and dephosphorylation reactions, respectively. Proteins separated by commas catalyze the same reaction. The steps in which the existence of an enzymatic reaction is uncertain are indicated by question marks.

The *myo*-inositol head group of the lipid phosphatidylinositol (PI) (Figure 1A) can be selectively phosphorylated at positions 3, 4 and 5 to generate seven unique species. These include three monophosphates -phosphatidylinositol 3 phosphate (PI3P), phosphatidylinositol 4 phosphate (PI4P) and phosphatidylinositol 5 phosphate (PI5P); three bisphosphates-phosphatidylinositol 4,5 bisphosphate [PI(4,5)P_2_], phosphatidylinositol 3,5 bisphosphate [PI(3,5)P_2_] and phosphatidylinositol 3,4 bisphosphate [PI(3,4)P_2_] and a single trisphosphate-phosphatidylinositol 3,4,5 trisphosphate [PI(3,4,5)P_3_] (Figure 1B). Biochemical studies across multiple cell types have revealed that phosphatidylinositol and the seven phosphoinositides are present in defined proportions (Figures 1C,D) and in many cases their cellular levels change in a precise and reversible manner during the response to specific cellular changes or environmental stimuli; these observations underscore their definition as important mediators of information transfer in cells. From a cell biological perspective, it is important to bear in mind that being lipids, phosphoinositides are not freely diffusible in the aqueous cytoplasm; therefore they are spatially restricted to the membrane at which they are produced and can move between intracellular compartments either by vesicular transport or through the action of lipid transport proteins (Cockcroft and Raghu, 2018). As a result of these constraints, each of the phosphoinositides has a distinct spatial pattern of distribution among organelles; most phosphoinositides are primarily enriched at one or two organelle membranes although minor pools of each lipid may also be found at other organelle membranes (Figure 2A) [reviewed in Di Paolo and De Camilli (2006), Balla (2013)]. In this review, we focus on the cellular functions of phosphoinositides in the nervous system. A comprehensive description of the cellular functions of these lipids has been published elsewhere and readers are referred to here for a detailed description of specific topics (Balla, 2013).

## Control of Cellular Phosphoinositide Levels

Within cells, phosphoinositides are generated through the selective addition or removal of phosphate groups from the *myo*-inositol headgroup. In general, the addition of phosphates is performed by an evolutionarily conserved group of enzymes called the phosphoinositide kinases. These enzymes are selective in two respects (i) They are specific for the substrate molecule on which they will act: for example a lipid kinase may act only on PI5P but not PI4P (ii) They are specific for the OH group on the *myo*-inositol ring at which they will add the phosphate group: for example an enzyme that will only add a phosphate at position 4 but not position 5. A large and evolutionarily conserved family of kinases has been described that show selectivity based on the above criteria (Table 1, Supplementary Table 1, and Figure 2B). The properties of these enzymes and the huge body of experimental analysis of these have been covered in an excellent and detailed review (Sasaki et al., 2009). Most enzymes of this family are conserved across all of eukaryota although some are seen only in metazoan genomes (e.g., Class I PI3K, PIP4K). In addition to the phosphoinositide kinases, lipid phosphatases that are able to selectively remove a phosphate group from the *myo*-inositol headgroup have been described. These enzymes also show substrate specificity and defined catalytic activity just like the phosphoinositide kinases (Table 1, Supplementary Figure 1, and Figure 2B) and (Sasaki et al., 2009) and are evolutionarily well conserved.

**TABLE 1:** Phosphoinositide kinase and phosphatase orthologs in *Drosophila melanogaster* and *Caenorhabditis elegans*.

	***H. sapiens***	***D. melanogaster***	***C. elegans***
**Kinases**			
	*PIK3C/CB/CD/CG*	PI3K92E/CG4141	PI3K/age-1
	*PIK3C2A/2B/2G*	PI3K68D/CG11621	PI3K/piki-1/NP_510529.1
	*PIK3C3*	PIK359F/vps34/CG5373/	PI3KC3/NP_001020954.1
	*PI4K2/2B*	PI4KIIA/CG2929	CELE_ZC8.6/NP_508849
	*PIK4CA/PI4KA*	PI4KIIIA/CG10260	CELE_Y75B8A.24/NP_499596.2
	*PIK4CB/PI4KB*	fwd/CG7004	–
	*PIP5K1A/1B/1C*	PIP5K59B/CG3682	ppk-1/NP_491576.2
	*PIP5K3/PIKFYVE*	Fab1/CG6355	–
	*PIP4K2A/2B/2C*	dpip4K/CG17471	ppk-2/NP_497500.1
**Phosphatases**			
*PI3-phosphatases*			
	*PTEN/TPTE2*	dPTEN/CG5671	hypothetical protein T07A9.6
	*MTM1/MTMR1/MTMR2*	dmtm/CG9115	MTMR1/NP_491531.2
	*MTMR3/MTMR4*	CG3632	MTMR3/NP_001022794.2
	*MTMR5/SBF1*	SBF/CG6939	MTMR5/NP_508888.2
	*MTMR6/MTMR7/MTMR8*	CG3530	MTMR6/NP_001022602.1
	*MTMR14*	–	–
*PI4-phosphatases*			
	*INPP4A/4B*	CG42271	INPP4/AAM97343.1
	*TMEM55A/55B*	CG6707	PI(4,5)P_2_ 4-phosphatase/NP_497624.3
	*SACM1L/SAC1*	sac1/CG9128	SAC1/NP_492518.2
*PI5-phosphatases*			
	*SYNJ1/2*	synj/CG6562	synaptojanin/NP_001023265.1
	*OCRL*	ocrl/CG3573	Inositol Polyphosphate-5-Phosphatase/NP_001255510.1
	*INPP5B*	–	–
	*INPP5J*	CG6805	–
	*SKIP/INPP5K*	CG9784	–
	*INPP5D/SHIP1*	–	–
	*INPPL1/SHIP2*	–	–
	*INPP5E*	INPP5E/CG10426	–
	*INPP5F/SAC2*	CG7956	CELE_W09C5.7/NP_001252206.1
	*FIG4*	Fig4/CG17840	CELE_C34B7.2/NP_492266.2
**Other components**			
	*PTPMT1*	ptpmt1/CG10371	CELE_F28C6.8/NP_001254162.1
Phospholipases			
	*PLCB1/2/3*	PLC/CG4574	–
	*PLCB4*	norpA/CG3620	1-phosphatidylinositol 4,5-bisphosphate phosphodiesterase/NP_001300035.1
	*PLCG1/G2*	Small wing/CG4200	Plc-3/NP_96205.2
	*PLCD1/3/4*	–	PI-PLC/NP_501213.1
	*PLCZ1*	–	–
	*PLCE1*	–	PLCE1/NP_001129926.1
PI transfer proteins			
	*PITPNA/NB*	Vibrator/CG5269	CELE_Y54F10AR.1/NP_497582.3
	*PITPNM1/NM2/NM3*	rdgB/CG11111	PITP/NP_497726.2
	*PITPNC1*	RdgB-beta/CG17818	–

*The orthologs of human phosphoinositide signaling genes (kinases, phosphatases. Phospholipases and lipid transfer proteins) in *D. melanogaster* and *C.elegans* have been reported. The orthologs have been obtained by the reciprocal blast approach, literature survey for reported orthologs or experimental evidence of a specific activity. The flybase gene IDs have been reported for *D. melanogaster* genes and NCBI gene IDs have been reported for *C. elegans* genes. (–) represents genes for which no orthologs are found in either genomes.*

Phospholipases are enzymes which belong to the class hydrolases, i.e., those that use a molecule of water to degrade substrates; they catalyze the breakdown of phospholipids into fatty acids and other constituent molecules (Dennis, 2015). They are named based on the position they hydrolyze on the backbone of phospholipids. Phospholipases are known to be involved in phospholipid turnover, membrane remodeling and neurotransmitter release in brain. Under pathological conditions they result in altered membrane permeability, ion homeostasis and accumulation of lipid peroxidases. The phospholipase of most relevance to phosphoinositide signaling is phospholipase C (EC 3.1.4.11) that is able to hydrolyze PI(4,5)P_2_ to generate inositol 1,4,5 trisphosphate (IP_3_) and diacylglycerol (DAG) (Rhee, 2001). The expression of phospholipase C genes varies across different brain compartments and tissues as detailed in this review. The phospholipases genes are well conserved across evolution (Table 1).

The phospholipid transfer proteins (LTPs) are molecules that facilitate the transfer of phospholipids between the membranes of sub-cellular compartments thus contributing to lipid homeostasis of cellular organelles. There are many classes of lipid transfer proteins; in the context of phosphoinositide signaling, the most important are the phosphatidylinositol transfer protein family that supports the transfer of PI from its site of synthesis, the endoplasmic reticulum, to the plasma membrane (Hsuan and Cockcroft, 2001). More recently additional classes of proteins such as the OSH/OSBP family have been discovered that appear to be required for PI4P transfer between organelle membranes (Cockcroft and Raghu, 2018). These lipid transfer proteins are distributed across a range of organisms (Table 1). Collectively the molecules described above are core components in the regulation of phosphoinositide signaling in eukaryotic cells (Balakrishnan et al., 2015) including those in the brain. The metabolism of each phosphoinositide and its cellular functions with respect to neurons are presented below.

## Cellular Functions of Phosphoinositides in the Nervous System

### PI3P

PI3P can be produced from PI by Class III PI3K (Vps34) in endosomes (Schu et al., 1993) or by class II phosphatidylinositol 3-phosphate kinase (PI3K) at the plasma membrane (Vanhaesebroeck et al., 2010). In addition, PI3P can be generated by dephosphorylation of PI(3,4)P_2_ by INPP4a (Sasaki et al., 2010) or PI(3,5)P_2_ by FIG4 (Chow et al., 2007). PI3P is mainly found localized at early endosomes and is involved in endosomal trafficking (Gaidarov et al., 2001) but is also generated at the autophagosomal membrane thus regulating autophagy (Noda et al., 2010).

With respect to neural cell function, PI3P may be particularly important in regulating the levels of cell surface receptors for neurotransmitters, or for the control of autophagy which is believed to be a key ongoing process in neurons that is relevant to neurodegeneration (Menzies et al., 2017). In hippocampal neurons of mice, PI3P was found to be localized at dendrites, axons and partially at synapses (Wang et al., 2011). The early endosomal pool of PI3P is involved in the post synaptic clustering of GABA receptors, thus regulating the strength of inhibitory post synapses in cultured hippocampal neurons (Papadopoulos et al., 2017). Depletion of Vps34, the key enzyme in PI3P generation, in selected brain regions results in neuronal degeneration and reactive gliosis that appear to be associated with defects in the endosomal system but not changes in autophagy (Zhou et al., 2010; Wang et al., 2011). Depletion of Vps34 in Schwann cells, the glia of the peripheral nervous system results in defective myelination associated with altered endo-lysosomal system and autophagy (Logan et al., 2017). Finally, PI3P has been found to be selectively deficient in the brains of human patients with Alzheimer’s disease (Morel et al., 2013) and mouse models of Alzheimer’s disease with associated defects in endosomal-lysosomal network [reviewed in Nixon (2017)].

### PI4P

PI4P is found in at least two major subcellular locations in cells, the Golgi complex and the plasma membrane. PI4P at Golgi coordinates several functions including vesicle trafficking, membrane biogenesis and lipid homeostasis (D’Angelo et al., 2008). At the Golgi apparatus, PI4P recruits proteins that bind this lipid and mediate its functions at this location thus regulating processes such as sphingolipid biosynthesis (Kawano et al., 2006) and cargo sorting. At the plasma membrane, PI4P is required for maintaining PI(4,5)P_2_ levels during receptor activated PLC signaling; PI4P can directly regulate the function of plasma membrane proteins such as KCNQ2/3 channels (Dickson et al., 2014) and smoothens the receptor for hedgehog signaling (Jiang et al., 2016). PI4P also binds to clathrin adaptors such as epsin R (Hirst et al., 2003) and AP-1 (Wang et al., 2003) thus regulating endosome trafficking. PI4P is generated by the phosphatidylinositol 4-kinase (PI4K) family of enzymes (Balla and Balla, 2006). At the plasma membrane PI4P is produced by the PI4KIIIα class of enzymes and regulates PLC dependent functions (Nakatsu et al., 2012; Balakrishnan et al., 2018). By contrast PI4P at the Golgi is generated by the PI4KIIIβ family (Godi et al., 1999; Walch-Solimena and Novick, 1999). PI4P can also be produced by dephosphorylation of PI(4,5)P_2_ by 5′ lipid phosphatases such as oculocerebrorenal syndrome of Lowe (OCRL) and Synaptojanin. These enzymes are likely to be particularly important in the context of neural cell function (*see below*). PI4P can also be degraded by the lipid phosphatase SACM1L (Sac1 in yeast), an ER resident enzyme that at plasma membrane endoplasmic reticulum contact sites (Stefan et al., 2011; Zewe et al., 2018).

In the nervous system, very little information is available on the direct effect of perturbations in PI4P levels, and more on the enzymes regulating it. The presence of PI4K2α at high concentration in synaptic vesicles indicates a direct or indirect role of PI4P in neuronal function, as a precursor of PI(4,5)P_2_ (Guo et al., 2003), which in turn has been identified to be crucial for clathrin mediated endocytosis (Wenk et al., 2001). *Pi4k2a* mutant adult mice lacking kinase activity exhibit progressive neurological disorders, including substantial degeneration of spinal axons (Simons et al., 2009). In *Drosophila* models, depletion of a protein complex that includes PI4KIIIα that generates PI4P at the plasma membrane has been shown to affect the neuronal accumulation Aβ_42_ oligomers (Zhang X. et al., 2017).

### PI5P

PI5P is the most recently discovered phosphoinositide (<10% of total cellular phosphatidylinositol monophosphates) (Rameh et al., 1997). Biochemical fractionation studies indicate that PI5P is distributed across multiple cellular compartments including the plasma membrane, nucleus, endo-lysosomal system and the Golgi (Sarkes and Rameh, 2010). A number of studies have suggested a role for PI5P in regulating chromatin function and transcriptional regulation in the nucleus [reviewed in Fiume et al. (2015)]. Ectopic expression of the *Shigella* phosphatase IpgD, that generates PI5P is known to cause endosomal sorting defects of the epidermal growth factor receptor (EGFR) (Ramel et al., 2011) and PI5P binding proteins have been identified in early endosomal factions (Boal et al., 2015). *In vitro*, PI5P is known to stimulate myotubularin (Schaletzky et al., 2003), proteins that play an important role in early endosomal sorting (Naughtin et al., 2010). Collectively these observations suggest that PI5P might regulate endosomal trafficking, but the mechanism remains unclear. The mechanism by which PI5P is generated remains unresolved. It has been argued that the enzyme PIKFYVE/Fab1 might synthesize PI5P from PI (Shisheva et al., 2015). An alternative model is that PI5P can be generated by the lipid phosphatase activity of the myotubularin family of enzymes acting on PI(3,5)P2 to generate PI5P in yeast (Walker et al., 2001) or mammalian cells (Vaccari et al., 2011). PI5P concentration can also be regulated by the phospholipid kinase phosphatidylinositol 5 phosphate 4-kinase (PIP4K) that is able to convert PI5P into PI(4,5)P_2_ (Gupta et al., 2013) and reviewed in Kolay et al. (2016).

There is limited information available on the role of PI5P in neurons. *In vitro* knock down or pharmacological inhibition of the PIP4K isoform PIP4K2C is reported to reduce mutant huntingtin protein aggregates by increasing basal autophagy, suggesting that the kinase could be a potential target for treatment of the progressive neurodegenerative Huntington’s disease (Al-Ramahi et al., 2017). *Drosophila* mutants with elevated PI5P levels have been reported to have defects in the early endosomal compartment of neurons (Kamalesh et al., 2017). It has also recently been reported that depletion of PIP4K2C results in defects in the recycling of Notch to the cell surface (Zheng and Conner, 2018); since Notch is a key regulator of neurogenesis, these findings may imply a role for PI5P, the substrate of PIP4K2C in brain development. Collectively these observations suggest a role for PI5P in regulating membrane transport in neural cells that remains to be fully understood.

### PI(4,5)P_2_

PI(4,5)P_2_ is mainly localized at the plasma membrane of cells although other minor pools of this lipid have been described on the membranes of internal organelles such as endosomes and lysosomes (van den Bout and Divecha, 2009). In this location it controls a number of cellular processes. Historically the oldest known function of PI(4,5)P_2_ is to serve as the substrate for receptor activated phospholipase C which generates inositol 1,4,5 trisphosphate (IP_3_) and diacylglycerol (DAG) which themselves serve as second messengers. However, it is now apparent that a major function of PI(4,5)P_2_ is to interact with and modulate the activity of numerous proteins that regulate key sub-cellular processes such as vesicular transport (endocytosis and exocytosis), cytoskeletal reorganization and the regulation of ion channel and transporter activity [reviewed in Kolay et al. (2016)]. PI(4,5)P_2_ is mainly produced by the phosphorylation of PI4P by phosphatidylinositol 4-phosphate 5-kinase (PIP5K) (Funakoshi et al., 2011). In humans, three PIP5K isozymes are expressed namely PIP5Kα, β, and γ (Funakoshi et al., 2011). A minor pool of PI(4,5)P_2_ is also synthesized by phosphatidylinositol 5-phosphate 4-kinase (PIP4K) using PI5P (Rameh et al., 1997) and PTEN using PIP_3_ as substrates (Rahdar et al., 2009). PI(4,5)P_2_ is consumed by the activity of PLC, Class I PI3K that converts it into PI(3,4,5)P_3_ and also by the activities of the 5’ phosphatases synaptojanin (SYNJ) and OCRL.

PI(4,5)P_2_ has a number of critical functions in the nervous system. Many receptors for neurotransmitters in the brain use G-protein coupled, PLC mediated hydrolysis of PI(4,5)P_2_ as a key step in signal transduction [reviewed in Dickson and Hille (2019)] and numerous ion channels in the nervous system require PI(4,5)P_2_ for their normal function [reviewed in Hille et al. (2015)]. In addition, PI(4,5)P_2_ regulates multiple steps of the synaptic vesicle cycle. The lipid kinase PIP5Kγ is enriched at nerve terminals and plays a key role in regulating PI(4,5)P_2_ levels (Wenk et al., 2001); PIP5Kγ^–/–^ mice show decreased PI(4,5)P_2_ in the brain and defects in synaptic transmission (Di Paolo et al., 2004). Dephosphorylation of PI(4,5)P_2_ is also important for the recycling synaptic vesicles that are endocytosed at the presynaptic terminal. The PI(4,5)P_2_ 5-phosphatase SYNJ1 plays a key role in the uncoating of clathrin-coated synaptic vesicles (Mani et al., 2007). Increased PI(4,5)P_2_ levels and clustering of clathrin-coated vesicles at nerve endings were observed in the neurons of SYNJ1-deficient mice (Cremona et al., 1999), and the internalization of postsynaptic AMPA receptor, involved in fast excitatory synaptic transmission, was inhibited in cultured *Synj1* knockout mouse hippocampal neurons (Gong and De Camilli, 2008). A role for PI(4,5)P_2_ has also been proposed for regulating cytoskeletal function in neurons; the kinesin motor Unc104 can bind PI(4,5)P_2_ (Klopfenstein et al., 2002) and this binding plays a key role in kinesin based transport in neurons (Klopfenstein and Vale, 2004; Kumar et al., 2010). Since PI(4,5)P_2_ performs multiple functions in neurons, an interesting question arises on how these are controlled independently. Recent studies have proposed a role for functional pools of PI(4,5)P_2_ generated in neurons by specific lipid kinases [(Chakrabarti et al., 2015) and reviewed in Kolay et al. (2016)].

### PI(3,5)P_2_

PI(3,5)P_2_ is a lipid that is primarily found on late endosomes and lysosomal membranes. Its levels rise in response to changes in extracellular stimuli including osmotic stress in yeast or stimulation with growth factors and phagocytosis in mammalian cells [reviewed in Hasegawa et al. (2017)]. A number of studies implicate PI(3,5)P_2_ in late endosomal dynamics and it has also been proposed to regulate the function of TPC ion channels on the lysosomal membrane (Jha et al., 2014). Tsuruta et al. (2009), Seebohm et al. (2012). PI(3,5)P_2_ is produced in cells by the activity of a PI3P-5 kinase (Fab1 in yeast and PIKFYVE in mammals). PI(3,5)P_2_ can be dephosphorylated *in vitro* by the activity of the phosphatase FIG4 to generate PI3P; members of the MTMR family have also been proposed to regulate PI(3,5)P_2_ levels although the relevance of these mechanisms *in vivo* remains unclear [reviewed in Hasegawa et al. (2017)].

In neurons decreased levels of PI(3,5)P_2_ directly affect NMDA-induced voltage-gated Ca^2+^ channel internalization which causes neuronal excitotoxicity (Tsuruta et al., 2009) and in the hippocampus neurons of new-born rats, defects in postsynaptic GluA1 receptor turnover are seen when PI(3,5)P_2_ levels are altered (Seebohm et al., 2012). PI(3,5)P_2_ levels have also been shown to increase during homeostatic downscaling, where neurons reduce their postsynaptic strength to prevent chronic neuronal hyperactivity (McCartney et al., 2014). A number of studies have shown a role for PI(3,5)P_2_ in regulating lysosomal function and autophagy in the nervous system. Analysis of spontaneous mouse mutants of *Fig4* and *Vac14*, members of the PIKFYVE-FIG4 enzyme complex, has shown that although these proteins are widely expressed across the body, the nervous system that is particularly susceptible to the loss of these enzymes, presumably through altered PI(3,5)P_2_ levels. These effects include neuronal degeneration, myelination defects and accumulation of inclusions in astrocytes [reviewed in Lenk and Meisler (2014)]. These findings have led to a mechanistic explanation for a number of human neurological syndromes in which proteins involved in PI(3,5)P_2_ metabolism are affected (see below).

### PI(3,4)P_2_

PI(3,4)P_2_ is a lipid that is primarily found at the plasma membrane and the early endosomal system. The function of this lipid is unclear but it has been proposed to regulate early endosomal dynamics and also alter the gain of the Class I PI3kinase signaling pathway (Zhang S. et al., 2017). The best characterized route of PI(3,4)P_2_ synthesis is the dephosphorylation of PI(3,4,5)P_3_ by 5′ phosphatases [reviewed in Hawkins and Stephens (2016)] although it has also been proposed to be generated in the early endosomal system during clathrin mediated endocytosis by Class II PI3K activity on PI4P (Posor et al., 2013; He et al., 2017). The myotubularin family of lipid phosphatases may control the levels of this lipid by degrading it to PI4P.

The available literature points to the role of PI(3,4)P_2_ in neurite initiation and dendrite morphogenesis (Zhang S. et al., 2017) by promoting actin aggregation at the site of initiation, leading to the cytoskeletal reorganization for forming the cylindrical neurite. PI(3,4)P_2_ was also shown to be present in the postmitotic multipolar neurons derived from radial glia. The actin remodeling protein lamellipodin binds to PI(3,4)P_2_ and recruits Ena/vasodilator-stimulated phosphoprotein (VASP) to positively regulate the number of primary processes in the multipolar cells (Yoshinaga et al., 2012).

### PI(3,4,5)P_3_

PI(3,4,5)P_3_ is a very low abundance lipid found at the plasma membrane of cells following activation of plasma membrane receptors (Ming et al., 1999; Henle et al., 2011; Arendt et al., 2014). The principal mechanism of synthesis is the activity of Class I PI3K enzymes that phosphorylate PI(4,5)P_2_ to generate PI(3,4,5)P_3_. PI(3,4,5)P_3_ can be degraded by the activity of 3′ phosphatases such as PTEN or by the action of 5′ phosphatases such as SHIP.

As in all other tissues and cell types, the levels of PI(3,4,5)P_3_ play a key role in growth through its ability to control cell division and size through activation of a number of intracellular signaling pathways (Engelman et al., 2006). PI(3,4,5)P_3_ has been found enriched in the growth cones of developing neurites (Ménager et al., 2004), and has been implicated in the control of numerous processes in both developing and mature neurons. These include remodeling of the actin cytoskeleton during neurite outgrowth and dendrite morphogenesis (Ming et al., 1999; Henle et al., 2011). Synthesis and availability of PI(3,4,5)P_3_ is also required for maintaining AMPA-type glutamate receptors at synaptic membrane, and this in turn regulates synaptic function in hippocampal neurons (Arendt et al., 2014). PTEN, that regulates PI(3,4,5)P_3_ levels is expressed and plays an important role during neuronal morphogenesis and differentiation (Lachyankar et al., 2000; van Diepen and Eickholt, 2008). Knockout of PTEN and resulting elevated PI(3,4,5)P_3_ level leads to an increase in the diameter of parallel fiber axons of granule cells, an increase in oligodendrocyte differentiation and *de novo* myelination of normally unmyelinated parallel fibers (Goebbels et al., 2017). Inhibition of PTEN by bisperoxovanadium has also shown to promote oligodendrocyte proliferation and myelination of dorsal root ganglion neurons (De Paula et al., 2014). At the same time, PTEN loss does not show an improvement in remyelination following brain injury (Harrington et al., 2010).

## Effectors of Phosphoinositide Signaling

The oldest known function of phosphoinositides is the regulation of calcium signaling. This process is triggered by the metabolic conversion of PI(4,5)P_2_ into IP_3_ and DAG by phospholipase C leading the activation of intracellular calcium release channels and plasma membrane calcium influx channels (Berridge and Irvine, 1989). However, we now know that phosphoinositides function through multiple mechanisms including their ability to bind to cellular proteins and regulate their activity. Given their negative charge, phosphoinositides may bind to proteins by interacting with single or clusters of positively charged residues (as in the case of KiR channels) (Hansen, 2015) or via well-defined protein domains (e.g., PH, PX, FYVE domains) [reviewed in Hammond and Balla (2015)]. A specific domain may be found in the context of multiple proteins that bind a specific phosphoinositide (for example identification of an FYVE domain may be an indication of a protein that binds PI3P and the context for its cellular function). More recently an unbiased quantitative mass spectrometry analysis has described a large set of more than 400 proteins that bind phosphoinositides (Jungmichel et al., 2014) and are likely to be key mediators of the effects of phosphoinositides in cells.

The well-characterized phosphoinositide-binding domains in effector proteins are the PH (pleckstrin homology), PX (phox homology), FYVE (Fab1, YOTB, Vac1 and EEA1), ENTH (epsin amino-terminal homology) and FERM (Four-point-one, ezrin, radixin and moesin) domains (Balla, 2005). Through their interactions with specific phosphoinositides, these effector proteins are recruited onto membrane surfaces and participate in various cellular processes (Di Paolo and De Camilli, 2006).

## Expression of Phosphoinositide Signaling Genes in the Human Nervous System

Gene expression in human tissues and cell types is differentially regulated depending on the developmental stage, tissue type, cell type and in some cases is altered in specific disease conditions. In addition to core genetic mechanisms, gene expression is also controlled epigenetically. Post-transcriptional processes can change the levels of transcripts and control of translation and protein stability can also affect the levels of proteins in the nervous system. The spatial and temporal pattern of gene expression can often give an insight into the potential function of a gene. Several studies have documented patterns of gene expression in human cells and tissues and an analysis of data on expression in brain regions, cell types and temporal patterns from such studies can provide an insight into the function of specific genes in the nervous system.

Several large-scale studies have documented gene expression in the human nervous system. Gene expression studies in the brain are mostly done from post-mortem samples. Studies have shown that death results in significant expression change in only 10% of genes with varied functional categorization, thus justifying the use of post-mortem brain samples for expression analysis (Franz et al., 2005). Such studies have included (i) the analysis of gene expression in specific cell types of the nervous system^1^, (ii) expression in distinct regions of the normal human brain and also a profile of the expression of each gene as a function of time starting with fetal development through to adult human brains and subsequently in the ageing brain^2^ and (iii) gene expression analysis in the diseased brain^3^. Likewise the Human Protein Atlas provides information on the sub-cellular localization and expression of more than 50% of human protein coding genes in major human tissues and organs (Bae et al., 2015). We have mined such databases for information on the expression of genes encoding components of the phosphoinositide signaling system in the human nervous system. The expression of genes in all these datasets is represented as TPM (Transcripts Per Million base pairs) values which is normalized for the dataset under study and cannot be compared across different datasets. These TPM values have been used to plot the heat maps and tables discussed below. The expression of genes in brain regions, cell types and their temporal expression profile is provided with a view to identifying those with potentially important roles in the structure and function of the nervous system.

### Spatial Expression Pattern of Phosphoinositide Signaling Genes in the Nervous System

**FIGURE 3:**
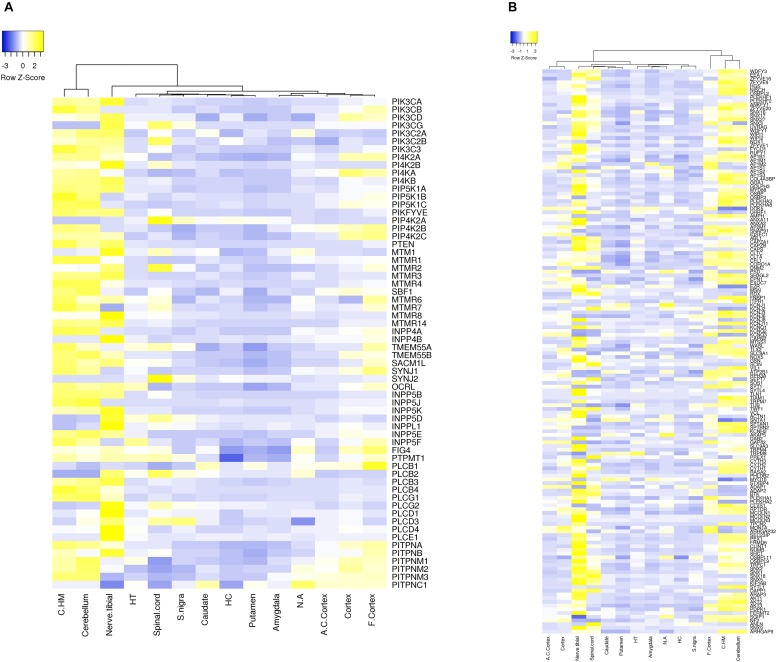
Expression of phosphoinositide signaling genes across human brain regions. A heat map showing the expression patterns. Data were extracted from the GTEx database. The expression values of genes for each of the brain region is normalized to the expression value of the gene in whole blood and the matrix thus obtained is represented as a heatmap. The normalized expression values range from –3 to 3 (Blue to Yellow). The map is clustered based on expression of genes in different regions of the Brain (across the column). The various brain regions represented are Cerebellar Hemisphere (C.HM), Cerebellum, Nerve tibial (N. tibial), Hypothalamus (HT), Spinal cord, Substantia nigra (S. nigra), Caudate, Hippocampus (HC), Putamen, Amygdala, Nucleus accumbens (N. accumbens), Anterior cingulate cortex (A. C. cortex), Cortex and Frontal cortex (F. cortex). Panel **(A)** shows the expression of kinases, phosphatases, phospholipases and lipid transfer protein while **(B)** shows the expression of phosphoinositide binding proteins. Individual proteins are indicated using HUGO nomenclature.

**FIGURE 4:**
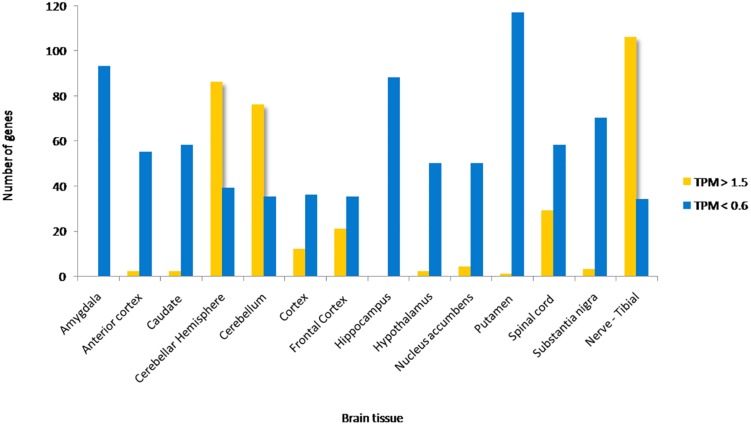
Proportion of phosphoinositide signaling genes with high and low expression in brain regions. The proportion of genes encoding members of the phosphoinositide signaling pathway with TPM values greater than 1.5 and less than 0.6 in specific brain regions are represented as a bar graph. Yellow bars represent TPM > 1.5, i.e., high expression genes and Blue bars represent TPM < 0.6, i.e., low expression genes.

Approximately 80–90% of protein-coding genes are expressed in some part of the brain during development and adult human life, though there are a large number of genes with a specific expression and alternate splicing patterns (Bae et al., 2015). Human specific expression (as compared to other primates) when noted is usually associated with enrichment in the neocortex and hippocampus. The expression values (TPM values) of 214 genes (phosphoinositide signaling genes listed in Table 1) in different brain regions was extracted from the GTEx database (see footnote 2) (Lonsdale et al., 2013). The expression of each gene was normalized to its expression in whole blood (TPM_brain_/TPM_whole_blood_) and is represented in the form of a heatmap that has been clustered across different regions of the brain (along the columns of the heatmap) (Figures 3A,B). This analysis reveals that most genes enriched in the nervous system are usually expressed in the cerebral cortex, cerebellum and tibial nerve. We then calculated the number of phosphoinositide signaling genes with TPM_brain_/TPM_whole_blood_ > 1.5 or TPM_brain_/TPM_whole_blood_ < 0.6 in each brain region in order to identify genes that are either highly expressed or minimally expressed in specific regions of the brain (Figure 4). This analysis revealed that the maximum number of genes with high TPM_brain_/TPM_whole_blood_ values were observed in peripheral nerves (represented by the tibial nerve), the cerebral and cerebellar hemispheres. In most of the other brain regions, the phosphoinositide signaling genes seem to be expressed at very low levels, with basal ganglia, hippocampus and amygdala having slightly higher numbers of such genes.

Of all the highly enriched genes in the brain, the 10 genes that have highest TPM values across various brain regions were noted. Of these, seven genes are highly expressed in at least 10 of the 15 major regions of the brain. These genes include *GAP43*, *KCNQ2*, *SNAP91*, *DOK 5*, *SH3GL2* and *SYT1*. GAP43 a growth-associated protein that is highly expressed during neuronal growth and axonal regeneration (Holahan, 2017); KCNQ2 a potassium channel which plays a key role in neuronal excitability (Jentsch, 2000); SNAP91, Synaptosome Associated Protein 91 a regulator of clathrin dependent endocytosis (Hill et al., 2001); DOK5 which interacts with phosphorylated receptor tyrosine kinases, activates MAP kinase signaling and is essential for neurite outgrowth (Grimm et al., 2001); SH3GL2 (SH3 Domain Containing GRB2 Like 2/, Endophilin A1) a known regulator of synaptic vesicle endocytosis (Vehlow et al., 2013) and SYT1(synaptotagmin 1) a regulator of neurotransmitter release at the synapse (18). There were also a number of highly expressed genes code for phosphoinositide binding proteins unique to the tibial nerve, i.e., not expressed in other parts of the nervous system (*FERMT2*, *FRMD6*, *SLC9A3*, *PLCE1*, *TRPC1*, *KCNJ8*, and *MCOLN3*) and may indicate a significant role for these in the function of peripheral nerves. It is interesting to note that most genes that show such enrichment patterns are phosphoinositide binding proteins or effectors, whereas genes encoding phosphoinositide metabolizing enzymes (kinases, phosphatases, lipases and transport proteins) appear to not show enrichment in a specific brain region, These findings are consistent with a broad role for phosphoinositide signaling in the nervous system with downstream effects being mediated in specific regions by proteins with more restricted expression patterns. While the data sets generated in such large scale gene expression analyses can be influenced by many factors, these data suggest the choices of tissue types for analyses as well as the potential nervous system compartments in which this signaling pathway could play a particularly important functional role in the context of normal physiology as well as diseases of the nervous system.

### Expression of Phosphoinositide Signaling Genes in Neural Cell Types

Every region of the nervous system includes multiple cell types including neurons, glial cells (astrocytes, microglia, oligodendrocytes) and endothelial cells of the vasculature. Transcriptomes of all these cell types have been generated and are available at http://www.brainrnaseq.org. More than 2000 genes seem to be differentially enriched in specific cell types (Zhang et al., 2016). Single cell transcriptome analysis shows that neurons express a higher number of genes as compared to other cell types and microglia and endothelial cells express fewest genes (Darmanis et al., 2015). Neuronal cells can be further grouped into seven sub-populations with differential gene expression patterns and gene-expression in distinct cell types has also shown to vary with age and under neuropathological conditions (Hagenauer et al., 2018).

**FIGURE 5:**
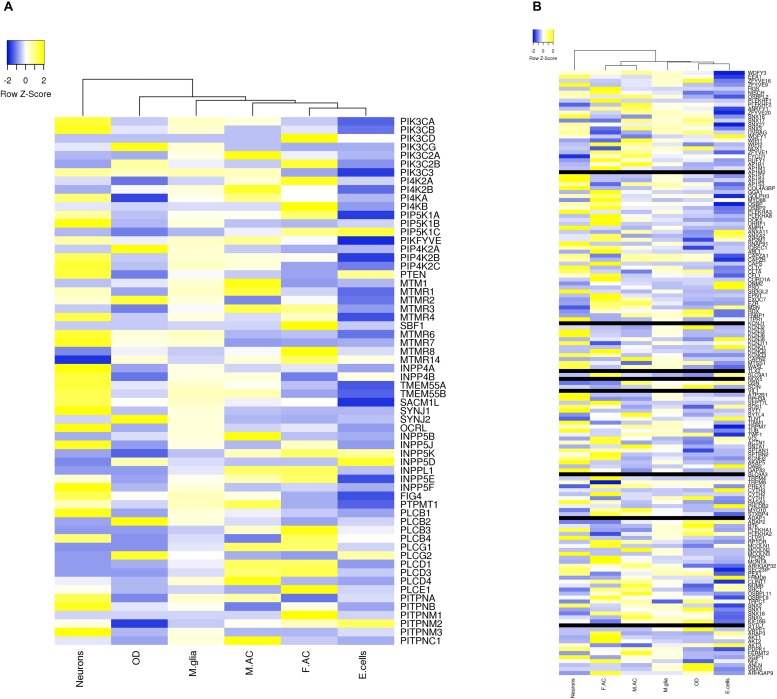
Expression level of phosphoinositide signaling genes in various brain cell types. The expression of genes involved in phosphoinositide signaling in specific neural cell types in human brain is shown. The data has been compiled from BrainRNAseq. The expression values range from –2 to 2 (Blue to Yellow). Black bar represents genes whose expression values were not available in the data set. The different cell types represented are Neurons, Oligodendrocytes (OD), Microglia (M.glia), Mature astrocytes (M.AC), Fetal astrocytes (F.AC) and Endothelial cells (E.cells). **(A)** Represents expression values of kinases, phosphatases, phospholipases and lipid transfer protein and **(B)** represents expression values of lipid binding proteins.

**FIGURE 6:**
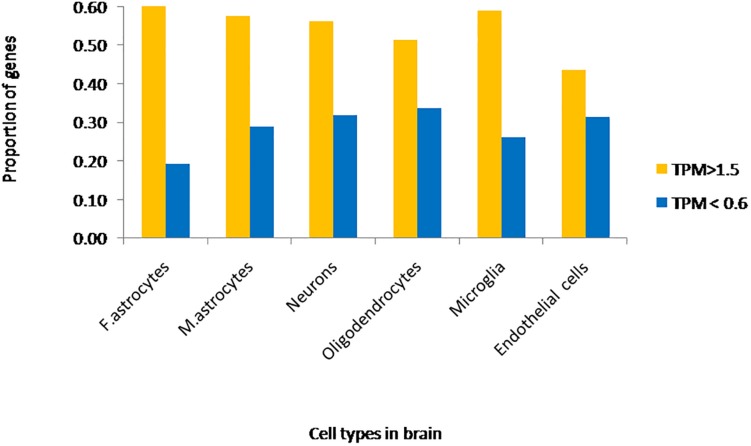
Fraction of phosphoinositide signaling genes with high and low expression in various cell types of the brain. The fraction of genes with TPM values greater than 1.5 and less than 0.6 in different neural cell types are represented in the graph. Yellow bars represent TPM > 1.5, i.e., high expression genes and Blue bars represent TPM < 0.6, i.e., low expression genes.

We extracted expression values (TPM values) for phosphoinositide signaling genes from Brain RNA-Seq data for various cell-types. The cell type specific expression values for 214 genes have been represented as a heatmap that has been clustered across different cell types in the brain (column-wise clustering in the heatmap) (Figures 5A,B). The probable connection between differential expression of genes and the disease condition has been discussed at the end of this section. The fraction of genes with TPM > 1.5 and TPM < 0.6 in each cell-type is presented (Figure 6). This analysis suggests that among all cell types, fetal astrocytes express the largest number of highly expressed genes (TPM > 1.5) and are also the cell type that show the smallest number of downregulated genes (TPM < 0.6). Among the various cell types, endothelial cells express the fewest number of differentially expressed phosphoinositide signaling genes. The top ten highly expressed genes for each cell-type were identified and compared for expression in multiple cell types. Of these AKT3, a serine/threonine protein kinase is highly expressed in all six cell types; AKT3, a mediator of growth factor signaling is implicated in a variety of biological processes such as cell proliferation, differentiation, apoptosis, tumorigenesis and glucose uptake. AKT3 is important for brain development and autophagy in neural cells (Howell et al., 2017; Soutar et al., 2018). Cofilin 1 (CFL1) is highly expressed in four of the cell-types (Fetal astrocytes, oligodendrocytes, microglia and endothelial cells). This protein is involved in polymerization and depolymerization of F-actin and G-actin in a pH dependent manner (Kanellos and Frame, 2016). CFL1 is required for neural tube morphogenesis and neural crest cell migration (Gehler et al., 2004). Clathrin heavy chain (CLTC) is also expressed in four of the cell types (neurons, oligodendrocytes, microglia and mature astrocytes). Clathrin is a major protein present in the cytoplasm that plays a role in vesicular transport and has been associated with neurological disorders (Nahorski et al., 2015). Spectrin alpha (SPTAN1), an essential cytoskeletal protein is expressed in the astrocytes, neurons and endothelial cells in the brain, and mutations in this gene is known to result in epileptic encephalopathy (Tohyama et al., 2015). Interestingly, among the most highly expressed genes, *SNAP91*, *PLCB1*, *SYNJ1* and *AP1S2* are all specific to neurons. Such expression patterns highlight the most useful cell type for the analysis of the cellular function of a specific gene. They could also suggest the cellular basis of a brain disorder when mutations are found in a given gene in human patients with brain disorders.

### Temporal Expression of Phosphoinositide Signaling Pathway Genes in the Brain

The BrainCloud application (see footnote 3) was used to obtain the expression (TPM values) of phosphoinositide signaling genes in the brain cortex of individuals with no neuropathological diagnosis during development (from fetal development to 80 years of age) (Colantuoni et al., 2011). Expression values were arranged as a function of increasing order of age and binned at every 5 years. As observed for all genes in the study (Colantuoni et al., 2011), significant changes in the expression of phosphoinositide signaling genes is seen immediately after birth, at adolescence and then at 70 years of age. Most enzymes in the phosphoinositide signaling cascade show limited temporal variation in expression. However, 20% of phosphoinositide metabolizing enzymes show deviations with expression values ranging from (0 to +1.5 or 0 to −1.5). A similar analysis on small set of genes has shown stable transcriptional networks and gene colocalization control PI metabolism in brain cortex during development (Rapoport et al., 2015). Thus, expression levels of such enzymes could be used as an indication of their potential role in the context of human disease.

### Gene Expression Data and Disease Relevance

Specific patterning of gene expression across different tissues and cell types in the brain is a feature observed in the expression data. Such expression patterns can be correlated well to some of the disease conditions of the brain as mentioned below. This data on expression could also be used to connect genes involved in PI signaling and their function in nervous system for a disease condition under study.

#### Phosphoinositide Kinases

Mutations in the genes *PIK3CA* and *PI4KA* that encode phosphoinositide kinases are associated with polymicrogyria and cortical dysplasia, disorders that result in altered cerebral cortex morphology and cellular composition (Mirzaa et al., 2012; Pagnamenta et al., 2015). Expression data shows that *PIK3CA* and *PI4KA* are highly expressed in the cerebral hemisphere compared to other brain regions and also enriched in the neurons as compared to other cell types in the brain. The high expression of these genes in the cerebral cortex tissue and a clear cortical development phenotype resulting from mutations in these genes underscores the potential value of using gene expression data to link gene function to disease phenotype in human brain disorders. A similar example is the *PIK3C3* gene that shows high expression in neurons; loss of this gene is associated with neuronal apoptosis and neurodegeneration in a mouse knockout model (Zhou et al., 2010). Loss of *PI4K2A* results in a progressive neurological motor disorder associated cerebellar and spinal cord degeneration (Simons et al., 2009) and this is well correlated with high expression of this gene in the cerebellum and cerebral cortex. Loss of *PIKFYVE* is associated with abnormal brain morphology and decreased brain weight (Zolov et al., 2012). This gene is highly expressed in mature astrocytes as compared to other cell types. Astrocytes are known to provide metabolic support to neurons and maintain brain morphology (Jha and Morrison, 2018) thus correlating the expression of this gene with phenotypes resulting from its depletion.

#### Phosphoinositide Phosphatases

Expression data shows that the *MTMR2* gene is upregulated in oligodendrocytes (cells required for myelination of axons in the CNS) and this is well-correlated with the finding that mutations in this gene can result in Charcot-Marie-tooth disease, type 4B1 (due to demyelination of the axons in the brain) (Bolino et al., 2000). Parkinson’s disease involves the slow degeneration of neurons in the substantia nigra area of the midbrain. The *INPP5F* gene that is linked by GWAS studies to Parkinson’s disease condition is differentially expressed in neurons (Nalls et al., 2014). Similarly the *PLCB1* gene, implicated in Alzheimer’s disease involving nerve cell damage and atrophy in the pre-frontal cortex seems to be enriched in this region (Bakkour et al., 2013). Overall these examples illustrate the value of expression data in trying to link human brain disease phenotypes with specific genes by studying their expression pattern in the brain.

## Phosphoinositide Signaling and Diseases of the Human Nervous System

Conceptually, disorders of the nervous system may arise from defects in processes that impact the development of the brain, the maintenance of normal function in developed brain cells and those that result in an inability to maintain the normal structure and function of the nervous system leading to neurodegenerative disease. There may of course be some overlap between these categories since it is now postulated that adult neurodegenerative diseases may arise from defects in brain development (Kovacs et al., 2014). Brain disorders can result from mutations in a single gene [Online Mendelian Inheritance in Man (OMIM)^4^ ] or DNA sequence variants in genes can alter the severity and clinical spectrum of a disorder. Defects in phosphoinositide signaling implicated in nervous system disorders of both these categories are discussed below. We include a brief description on the key clinical features of each disease; comprehensive descriptions on each neurological disorder can be found in Brain’s Diseases of the Nervous system (Donaghy, 2011).

### Monogenic Disorders

#### Brain Development Disorders

Dominant or recessive single-gene mutations in proteins regulating phosphoinositide signaling have been implicated in neurodevelopmental disorders. The oldest and most well-known, that impacts neurodevelopment, is the X-linked monogenic disorder OCRL, or Lowe syndrome. Lowes syndrome results from mutations in the *OCRL* gene, that encodes one of the inositol polyphosphate 5-phosphatase enzymes that dephosphorylates PI(4,5)P_2_ to generate PI4P [(Olivos-glander et al., 1995) and reviewed in Mehta et al. (2014)]. The disease affects the central nervous system along with the eyes and kidneys, although the extent to which each organ is affected is variable. With regard to the nervous system, most patients show varying degrees of developmental delay, intellectual disability, hypotonia, absence of deep tendon reflexes and convulsions. Motor development is affected, and many patients show moderate mental retardation. Maladaptive behaviors including stubbornness and temper tantrums have been reported (Kenworthy et al., 1993; Bökenkamp and Ludwig, 2016). PI(4,5)P2 is known to control a number of key cellular processes in developing neural cells particularly in relation to endocytosis and the control of plasma membrane receptor composition and alterations in these may result in abnormal brain function. However, the basis for nervous system defects remains unknown and the cellular mechanism by which OCRL deficiency results in neural cell dysfunction also remains to be investigated.

Monogenic mutations in members of the Class I PI3K pathway has been found to cause multiple disorders affecting brain development in addition to non-neural consequences. Many of these mutations occur as somatic mosaics and the extent of defects in brain development depend on the stage of embryonic development at which the mutation occurs (Madsen et al., 2018). A comprehensive study on 33 children with pediatric epilepsy identified germline mutations (*PTEN*) or mosaic activating mutations (*PIK3CA* and *AKT3*) in PI3K pathway genes which dramatically manifests as different forms of brain malformations like megalencephaly and cortical dysplasia (Poduri et al., 2012; Jansen et al., 2015). Phosphatase and tensin homolog (*PTEN*) gene codes for a phosphatidylinositol 3,4,5 trisphosphate 3-phosphatase which negatively regulates PI3K/AKT pathway (Sansal and Sellers, 2014). Dominant mutations in *PTEN* are associated with macrocephaly autism syndrome, extreme macrocephaly ranging from >2.5 to 8.0 SD above the mean (Butler et al., 2005; Varga et al., 2009; Mcbride et al., 2010), poorly developed white matter and reduced cognitive abilities (Frazier et al., 2015). Autosomal mutations in *PIK3CA*, coding for p110α subunit of Class I phosphatidylinositol 3-kinase (PI3K), results in megalencephaly-capillary malformation-polymicrogyria syndrome which is characterized by brain overgrowth (megalencephaly), capillary malformations, and thick cerebral cortex due to development of excessive, unusually small folds on brain surface (polymicrogyria) (Mirzaa et al., 2012). And finally, mosaic mutation at p.Glu17Lys in pleckstrin homology (PH) domain of AKT3, a predominant effector of PI3K signaling, causes an elevation in binding to phosphatidylinositol-3,4-bisphosphate, with patients exhibiting asymmetric cortical dysplasia, while constitutive mutation in other domains showed a range of brain malformations (Poduri et al., 2012; Alcantara et al., 2017). Such overgrowth phenotypes in the brain are likely to arise from the key role that PI(3,4,5)P_3_ plays in the control of cell proliferation and cell growth.

Polymicrogyria is also a symptom in disorders resulting from *PIK4CA* (phosphatidylinositol 4-kinase), *FIG4* (phosphoinositide 5-phosphatase) and *AKT3* mutations (Baulac et al., 2014; Pagnamenta et al., 2015), while mutations in the 5-phosphatase domain of polyphosphate 5-phosphatase *INPP5E* manifests as the ciliopathy Joubert Syndrome 1 (Bielas et al., 2009) characterized by cerebellar hypoplasia/aplasia (incomplete development of cerebellum), thickened cerebellar peduncles and abnormally large interpeduncular fossa (‘molar tooth sign’) (Travaglini et al., 2013; Shetty et al., 2017). These observations illustrate the function phosphoinositides in brain development; a key biochemical mechanism is likely to be the role of PI(4,5)P2 and PI(3,45)P3 in cell division and growth.

#### Neurodegeneration

Phosphoinositides play an active role in membrane trafficking and cellular signaling. Since neurons are terminally differentiated cells, they are sensitive to cellular stress and susceptible to cell death. Any perturbation in the tightly regulated levels of phosphoinositides affects intracellular vesicular trafficking, membrane turnover and may result in the accumulation of cellular components that should have been degraded resulting ultimately in neuronal degeneration. Such alterations in phosphoinositide levels in the brain can result from mutations in enzymes that regulate their levels or drugs that inhibit the activity of enzymes involved in PI signaling. Important examples of such mutations are discussed below.

Synaptojanin 1 (SYJN1), a polyphosphoinositide phosphatase whose Sac1 domain dephosphorylates PI(4,5)P_2_ and PI(3,4,5)P_3_ (Cremona et al., 1999), is highly concentrated at nerve terminals (McPherson et al., 1996). Two distinct disorders have been associated with mutations in *SYNJ1* gene. Alterations in SYNJ1 likely alter PI(4,5)P_2_ at the synapse, influence the synaptic vesicle cycle and hence contribute to the brain phenotypes described in human patients. Studies by independent groups identified homozygous missense mutation at R258Q of SYNJ1 in patients with early onset Parkinson disease-20 that affect the phosphatase activity of Sac1 domain (Krebs et al., 2013; Quadri et al., 2013; Olgiati et al., 2014). The patients exhibited tremor and bradykinesia with mild cerebral cortical atrophy. At the same time, mutations resulting in complete loss of SYNJ1 function have been identified in patients with early infantile epileptic encephalopathy 53, a severe neurodegenerative disorder characterized by epileptic seizures, severe intellectual disability and spastic quadriplegia (Hardies et al., 2016; Al Zaabi et al., 2018). A homozygous truncating mutation in *SYNJ1* identified in a patient with intractable seizures also showed neurofibrillary degeneration and presence of tau protein in substantia nigra region of brain (Dyment et al., 2015). In addition to specific mutations in SYNJ1, trisomy at the locus 21q22.11 containing *SYNJ1* gene has been identified in multiple lymphoblastoid cell lines developed from individuals with Down syndrome; these cells show enlarged endosomes as a result of overexpression of SYNJ1 in these cells (Cossec et al., 2012), indicating a link of SYNJ1 to Down syndrome. This result was substantiated from previous observations of increased SYNJ1 expression in brains of patients with Down syndrome (Arai et al., 2002), as well in Ts65Dn mice, a mouse model for Down syndrome exhibiting altered PI(4,5)P_2_ levels (Voronov et al., 2008). It is noted that patients with Down syndrome show early onset Alzheimer’s disease; this could result from a combination of overexpression of APP, the precursor of the Aβ peptide and overexpression of SYNJ1, which results in decreased levels of PI(4,5)P_2_, altered cellular handling of the Aβ peptide and hence early onset disease. Overall these findings imply that the specific phenotype in the patient may be impact by the type of mutation in a given gene and its effect on the activity of the enzyme it encodes as well as interactions with other genetic changes in the patient’s genome leading to altered disease phenotype.

FIG4 is another Sac domain-containing phosphoinositide 5′-phosphatase that dephosphorylates PI(3,5)P_2_ to PI3P. ‘Pale tremor mouse’ that carries a mutation in the *Fig4* gene shows enlarged late-endosomes/lysosomes with severe neurodegeneration of dorsal root ganglion cells and large myelinated axons. The mice exhibit severe tremor and impaired motor coordination resembling Charcot-Marie-Tooth disorder in humans (Chow et al., 2007) and has been widely used as a model to study neurodegenerative disorders associated with *FIG4* mutations. Compound-heterozygous loss-of-function mutation and I41T/R183X missense mutation have been identified in the autosomal recessive Charcot-Marie-Tooth type 4J (CMT4J) syndrome (Chow et al., 2007) with patients exhibiting progressive, asymmetric motor neuron degeneration, severe demyelination and axonal loss (Zhang et al., 2008; Nicholson et al., 2011). Individuals carrying one *FIG4* null allele or missense mutation in one allele, and a normal allele, exhibit one form of autosomal dominant amyotrophic lateral sclerosis (Chow et al., 2009), with less severe symptoms to CMT4J. In contrast, null mutations in *FIG4* resulting in complete loss of function is the cause of Yunis-Varon syndrome, which is characterized by severe neurological impairment affecting central nervous system with enlarged vacuoles in neurons, muscle and cartilage, in addition to skeletal abnormalities (Campeau et al., 2013; Nakajima et al., 2013). Around 30 individuals have thus far been identified with this syndrome (Campeau et al., 2013). PI3P is a lipid that controls endosomal trafficking and autophagy. Thus, phenotypes resulting from *FIG4* and *MTMR* mutations likely affect the brain through alterations in membrane turnover in neural cells.

Charcot-Marie-Tooth type 4B is a group of autosomal recessive demyelinating neuropathic disorders that are typically characterized by irregular thickness of myelin sheaths due to abnormal outfolding (Othmane et al., 1999). Mutations in myotubularin related protein 2 (*MTMR2*), which dephosphorylates PI3P and PI(3,5)P_2_ and the catalytically inactive pseudophosphatase *MTMR13*/*SBF2* (SET binding factor 2) genes are responsible for CMT4B1 and CMT4B2, respectively (Bolino et al., 2000; Azzedine et al., 2003). Even though MTMR13 is catalytically inactive, it has been suggested that the protein associates with MTMR2 to form a membrane-associated complex to regulate phosphoinositide levels (Robinson and Dixon, 2005). Since Both FIG2 and MTMR2 regulate the PI(3,5)P_2_ pool in the cells; the myelin sheath folding abnormalities could be due to the vesicle trafficking defects in neurons or Schwann cells as a result of PI(3,5)P_2_ dysregulation.

Mutations in other phosphoinositide signaling genes have been implicated in less severe forms of neuropathy and intellectual disability. In addition to the developmental disorder Joubet Syndrome, mutations in 5′-phosphatase inositol polyphosphate 5-phosphatase E (*INPP5E*) causes moderate mental retardation, truncal obesity, retinal dystrophy, and micropenis (MORM) syndrome (Hampshire et al., 2006). Similarly, mutations in 5’-phosphatase inositol polyphosphate 5-phosphatase K (*INPP5K*) result in the autosomal recessive MDCCAID (Muscular Dystrophy, Congenital, Cataracts And Intellectual Disability) disorder, where the patients suffer from mild intellectual disability in addition to the main symptoms of muscular dystrophy and early-onset cataract (Osborn et al., 2017; Wiessner et al., 2017). Table 2 lists nervous system disorders associated with mutations in phosphoinositide signaling genes. Since a monogenic disease cannot be studied experimentally in humans, several mouse strains with specific gene knockouts have been generated. Mouse knockouts of genes involved in phosphoinositide pathway with their phenotypes collated from Mouse Genome Informatics database^5^ are summarized in Supplementary Table 1.

**TABLE 2:** Monogenic disorders due to mutations in phosphoinositide signaling genes.

**Gene name**	**Ensembl ID**	**Chromosome location**	**Gene/locus MIM number**	**Phenotype**	**Phenotype MIM number**	**Inheritance**
**Brain growth and development**						
*PIK4CA/PI4KA*	ENSG00000241973.6	22q11.21	600286	Polymicrogyria, perisylvian, with cerebellar hypoplasia and arthrogryposis	616531	Autosomal recessive
*PIK3CA*	ENSG00000121879.3	3q26.32	171834	Megalencephaly-capillary malformation-polymicrogyria syndrome, somatic	602501	
*PTEN*	ENSG00000171862.5	10q23.31	601728	Macrocephaly/autism syndrome	605309	Autosomal dominant
*OCRL*	ENSG00000122126.11	Xq26.1	300535	Lowe syndrome	309000	X-linked recessive
*INPP5E*	ENSG00000148384.11	9q34.3	613037	Joubert syndrome 1	213300	Autosomal recessive
*FIG4*	ENSG00000112367.6	6q21	609390	Polymicrogyria, bilateral temporooccipital	612691	Autosomal recessive
*WDFY3*	ENSG00000163625.11	4q21.23	617485	Microcephaly 18, primary, autosomal dominant	617520	Autosomal dominant
*AKT3*	ENSG00000117020.12	1q43-q44	611223	Megalencephaly-polymicrogyria-polydactyly- hydrocephalus syndrome 2	615937	Autosomal dominant
(*1*) *NF2*	ENSG00000186575.13	22q12.2	607379	Meningioma, NF2-related, somatic	607174	Autosomal dominant
(*2*) *NF2*	ENSG00000186575.13	22q12.2	607379	Neurofibromatosis, type 2	101000	Autosomal dominant
(*3*) *NF2*	ENSG00000186575.13	22q12.2	607379	Schwannomatosis, somatic	162091	
**Neurodegeneration**						
*SYNJ1*	ENSG00000159082.13	21q22.11	604297	Parkinson disease 20, early-onset	615530	Autosomal recessive
(*1*) *FIG4*	ENSG00000112367.6	6q21	609390	Yunis-Varon syndrome	216340	Autosomal recessive
(*2*) *FIG4*	ENSG00000112367.6	6q21	609390	Amyotrophic lateral sclerosis 11	612577	Autosomal dominant
*ANXA11*	ENSG00000122359.13	10q22.3	602572	Amyotrophic lateral sclerosis 23	617839	Autosomal dominant
(*1*) *ITPR1*	ENSG00000150995.13	3p26.1	147265	Gillespie syndrome	206700	Autosomal dominant/recessive
(*2*) *ITPR1*	ENSG00000150995.13	3p26.1	147265	Spinocerebellar ataxia 15	606658	Autosomal dominant
(*3*) *ITPR1*	ENSG00000150995.13	3p26.1	147265	Spinocerebellar ataxia 29, congenital non-progressive	117360	Autosomal dominant
(*1*) *SPTBN2*	ENSG00000173898.7	11q13.2	604985	Spinocerebellar ataxia 5	600224	Autosomal dominant
(*2*) *SPTBN2*	ENSG00000173898.7	11q13.2	604985	Spinocerebellar ataxia, autosomal recessive 14	615386	Autosomal recessive
**Peripheral neuropathy**						
*MTMR2*	ENSG00000087053.14	11q21	603557	Charcot-Marie-Tooth disease, type 4B1	601382	Autosomal recessive
*MTMR13/SBF2*	ENSG00000133812	11p15.4	607697	Charcot-Marie-Tooth disease, type 4B2	604563	Autosomal recessive
*MTMR5/SBF1*	ENSG00000100241.16	22q13.33	603560	Charcot-Marie-Tooth disease, type 4B3	615284	Autosomal recessive
*FIG4*	ENSG00000112367.6	6q21	609390	Charcot-Marie-Tooth disease, type 4J	611228	Autosomal recessive
*DNM2*	ENSG00000079805.12	19p13.2	602378	Charcot-Marie-Tooth disease, axonal type 2M	606482	Autosomal dominant
*DNM2*	ENSG00000079805.12	19p13.2	602378	Charcot-Marie-Tooth disease, dominant intermediate B	606482	Autosomal dominant
**Epilepsy/seizure**						
(*1*) *KCNQ2*	ENSG00000075043.13	20q13.33	602235	Epileptic encephalopathy, early infantile, 7	613720	Autosomal dominant
(*2*) *KCNQ2*	ENSG00000075043.13	20q13.33	602235	Seizures, benign neonatal, 1	121200	Autosomal dominant
(*3*) *KCNQ2*	ENSG00000075043.13	20q13.33	602235	Myokymia	121200	Autosomal dominant
*KCNQ3*	ENSG00000184156.11	8q24.22	602232	Seizures, benign neonatal, 2	121201	Autosomal dominant
*PLCB1*	ENSG00000182621.12	20p12.3	607120	Epileptic encephalopathy, early infantile, 12	613722	Autosomal recessive
*SYNJ1*	ENSG00000159082.13	21q22.11	604297	Epileptic encephalopathy, early infantile, 53	617389	Autosomal recessive
**Mental retardation**						
*INPP5E*	ENSG00000148384.11	9q34.3	613037	Mental retardation, truncal obesity, retinal dystrophy, and micropenis	610156	Autosomal recessive
*AP1S2*	ENSG00000182287.9	Xp22.2	300629	Pettigrew syndrome/Mental retardation, X-linked syndromic 5	304340	X-linked recessive
*COL4A3BP*	ENSG00000113163.11	5q13.3	604677	Mental retardation, autosomal dominant 34	616351	Autosomal dominant
*CLTC*	ENSG00000141367.7	17q23.1	118955	Mental retardation, autosomal dominant 56	617854	
*SKIP/INPP5K*	ENSG00000132376.15	17p13.3	607875	Muscular dystrophy, congenital, with cataracts and intellectual disability	617404	Autosomal recessive
*PIP5K1C*	ENSG00000186111.4	19p13.3	606102	Lethal congenital contractual syndrome 3	611369	Autosomal recessive

*Table representing monogenic brain disorders reported as loss of function or mutation in genes of the phosphoinositide signaling pathway. The reaction catalyzed by each gene product and its function in brain are also reported.*

### Phosphoinositide Signaling in Bipolar Disorder

Bipolar disorder (BD) is a psychiatric illness characterized by disruptive mood swings resulting in alterations between mania and depression (Simon, 2003; Tighe et al., 2011). Studies in the early 1990s showed elevated levels of phosphatidylinositol-4, 5-bisphosphate (PIP_2_) in platelets from BD patients compared to controls (Soares and Mallinger, 1995) and that Protein kinase C (PKC) activity is enhanced in BD patients. These observations are consistent with a hyperactive PI cycle (Friedman et al., 1993) in BD patients. Although it was John Cade who first had treated manic depression with Lithium bromide (Cade, 1949) an understanding of the therapeutic action of lithium (Li^+^) came from the studies of Allison and Stewart who discovered that Li^+^ decreases the concentration of *myo*-inositol in the cerebral cortex of rats (Berridge et al., 1982, 1989) and that the decrease in *myo*-inositol concentration upon Li^+^ treatment is accompanied by an increase in the concentration of inositol monophosphate suggesting that Li^+^ might inhibit the enzyme inositol monophosphatase (IMPase) (Berridge et al., 1982; Harwood, 2005). Elevation of the intracellular inositol monophosphate level upon Li^+^ treatment was also observed in the salivary glands in insects (Berridge et al., 1982). IMPase dephosphorylates inositol 1-monophosphate to generate free *myo*-inositol and thereby replenish intracellular myo-inositol pool (Ohnishi et al., 2007; Agam et al., 2009). *Myo*-inositol is a precursor of phosphatidylinositol and when condensed with CDP-diacylglycerol by the enzyme phosphatidylinositol synthase generates phosphatidylinositol. Thus, IMPase could be integral to the maintenance of the phosphoinositide pools in mammalian cells. Such observations led Berridge to propose the “inositol depletion hypothesis” which suggests that Li^+^ prevents the recycling of *myo*-inositol by inhibiting the enzyme IMPase thereby preventing re-synthesis of phosphatidylinositol and phosphatidylinositol-4, 5-bisphosphate (PIP_2_) synthesis (Berridge et al., 1989). Purified IMPase was subsequently shown to be inhibited by Li^+^ (Hallcher and Sherman, 1980) via a non-competitive mechanism. Li^+^ binds to the enzyme-substrate complex, displacing Mg^2+^ from the active site thereby preventing the hydrolysis of the phosphate from inositol monophosphate (Harwood, 2005). However, decisive evidence that inositol depletion and alteration in PIP_2_ levels underlie the therapeutic action of Li^+^ in BD patients remains to be established and it has also recently been proposed that elevated inositol monophosphate levels may underlie the mechanism of action of Li^+^ (Saiardi and Mudge, 2018). Although a recent elegant biochemical analysis has once again established the ability of Li^+^ to regulate phosphatidylinositol turnover in neurons, the mechanism by which it does so and the relevance to therapeutic effects in BD remains a topic of active investigation (Mertens et al., 2015). Apart from IMPase, Li^+^ has multiple additional targets including the inositol transporter SMIT1, cyclooxygenase (COX), beta-arrestin 2 (βArr2) and glycogen synthase kinase 3 (GSK-3) (Freland and Beaulieu, 2012). The relative importance of these additional targets and their relation to PI turnover in the brain remains to be investigated.

### Phosphoinositide Signaling Gene Loci Linked to Neurological Disorders

Identification of genetic variants associated with specific complex phenotypes for the brain have been made possible through the use of genome-wide association studies (GWAS) (Ripke et al., 2014; Ikeda et al., 2018). This approach does away with the bias of candidate gene approach where genes to be analyzed are selected based on pre-existing knowledge regarding their biological relevance. GWAS have generated huge amount of information spanning the entire genome to identify single nucleotide polymorphisms (SNPs) and other genomic variants associated with brain disorders. A catalog of such variants is curated and maintained by NHGRI-EBI^6^ (MacArthur et al., 2017) and can be mined to identify variants associated with genes involved in phosphoinositide signaling.

Genes encoding a number of phosphoinositide kinases and phosphoinositide binding proteins have been linked in GWAS studies with brain disorders. For example, variants in the kinase *PIK3C2A*, and the PI binding proteins *UHRF1* (PI5P), *SNAP91* and *CAPN2* [PI(4,5)P_2_], *RPTOR* [PI(3,5)P_2_], *AKT3* [PI(3,4)P_2_ and PI(3,4,5)P_3_] have been linked to schizophrenia patients (Goes et al., 2015) and another study identified *PIK3C2A* linked to schizophrenia and bipolar disorder (Ruderfer et al., 2014). In other studies, associations have been proposed between *PIK4CA* and *PIP4K2A* (previously named PIP5K2A) and schizophrenia (Schwab et al., 2006; Jungerius et al., 2008; Thiselton et al., 2009). Association studies have also suggested a link between PIP4K2A and a protective effect in tardive dyskinesia, which occurs as a complication of long-term anti-psychotic treatment (Fedorenko et al., 2015), while the diplotype ATTGCT/ATTGCT in *PIP4K2A* results in poor antipsychotic response in schizophrenia patients (Kaur et al., 2014). Another GWAS study has associated a *PI4K2B* SNP with cannabis dependence (Sherva et al., 2016), which in turn is associated with increased risk of schizophrenia (Vaucher et al., 2017), while *PI4K2B* has also been identified as a candidate gene in schizophrenia on a study on Scottish population cohort (Houlihan et al., 2009).

Mirroring the numerous monogenic disorders that result from mutations in phosphoinositide phosphatases, GWAS studies have linked mutations in the 5-phosphatase gene *SYNJ1* causing PARK20 to Alzheimer’s disease (Herold et al., 2016), while another study has directly correlated SYNJ1 polymorphisms to age of onset in familial Alzheimer’s disease (Miranda et al., 2018). In a large study conducted in 74,026 individuals, a new locus was also identified with SNPs in *INPP5D*/*SHIP1*, the enzyme dephosphorylating PI(3,4,5)P_3_ into PI(3,4)P_2_, linking it to Alzheimer’s disease (Lambert et al., 2013). SNPs in genes for the 4-phosphatase INPP4B and the 5-phosphatase INPP5B have been linked to sporadic amyotrophic lateral sclerosis (Xie et al., 2014). An intronic variant was identified in the genetic locus of myotubularin related protein 7 (MTMR7), a 3-phosphatase dephosphorylating PI3P and inositol 1,3-bisphosphate and linked to variant Creutzfeldt-Jakob disease susceptibility (Sanchez-Juan et al., 2012).

Although the detection of a disease susceptibility locus is by no means a validated proof implicating a gene in a brain disorder, it can provide a starting point for validation of the mechanistic role of that gene through other forms of genetic analysis. Table 3 depicts the genetic loci in phosphoinositide signaling genes that are linked to brain disorders in humans.

**TABLE 3:** GWAS and other association studies linking phosphoinositide signaling genes to brain disorders.

**Gene name**	**PubMed ID**	**Disease/Trait**	**Region**	**Strongest SNP-risk allele**	**Type of variant**
**Kinases**					
(*1*) *PIK3C2A*	26198764	Schizophrenia	11p15.1	rs2008905-T	intron_variant
(*2*) *PIK3C2A*	24280982	Schizophrenia or bipolar disorder	11p15.1	rs4356203-?	intron_variant
(*3*) *PIK3C2A*	21926974	Schizophrenia	11p15.1	rs4356203-?	intron_variant
*PIK3C2G*	24086445	Gray matter volume (schizophrenia interaction)	12p12.3	rs11044045-?	intron_variant
*PI4K2B*	27028160	Cannabis dependence	4p15.2	rs73252553-A	non-_coding_transcript_exon_variant
*PIP4K2C*	18794853	Rheumatoid arthritis	12q13.3	rs1678542-C	intron_variant
**Phosphatases**					
***PI3-phosphatases***					
*MTMR3*	28247064	Cerebrospinal P-tau181p levels	22q12.2	rs41157-T	non-_coding_transcript_exon_variant
*MTMR4*	25644384	Cognitive function	17q22	rs2429369-?	intron_variant
*MTMR7*	22137330	Creutzfeldt-Jakob disease (variant)	8p22	rs4921542-?	intron_variant
***PI4-phosphatases***					
*INPP4A*	23092984	Bipolar disorder with mood-incongruent psychosis	2q11.2	rs12617721-C	intron_variant
*INPP4B*	24529757	Amyotrophic lateral sclerosis (sporadic)	4q31.21	rs2667100-?	intron_variant
*SACM1L/SAC1*	22041458	Response to anti-depressant treatment in major depressive disorder	3p21.31	rs2742417-T	5_prime_UTR_variant
***PI5-phosphatases***					
*SYNJ1*	26830138	Alzheimer disease and age of onset	21q22.11	rs147991290-T	intron_variant
(*1*) *INPP5B*	24529757	Amyotrophic lateral sclerosis (sporadic)		kgp15327256-?	
(*2*) *INPP5B*	24390342	Rheumatoid arthritis	1p34.3	rs28411352-T	3_prime_UTR_variant
*INPP5D/SHIP1*	24162737	Alzheimer’s disease (late onset)	2q37.1	rs35349669-T	intron_variant
*INPP5F/SAC2*	25064009	Parkinson’s disease	10q26.11	rs117896735-A	intron_variant
**Phospholipases**					
***Phospholipase C***					
(*1*) *PLCB1*	24564958	Social communication problems	20p12.3	rs3761168-A	intron_variant
(*2*) *PLCB1*	20125193	Cognitive performance	20p12.3	rs6118083-?	intron_variant
(*3*) *PLCB1*	19734545	Cognitive performance	20p12.3	rs6056209-?	intron_variant
(*4*) *PLCB1*	26079190	Suicide ideation score in major depressive disorder	20p12.3	rs6055685-A	intron_variant
(*5*) *PLCB1*	27846195	Response to paliperidone in schizophrenia (Multivariate)	20p12.3	rs6055808-?	intron_variant
(*1*) *PLCB2*	21926974	Schizophrenia	15q15.1	rs1869901-?	intron_variant
*2) PLCB2*	25056061	Schizophrenia	15q15.1	rs56205728-A	intron_variant
**PI transfer proteins**					
(*1*) *PITPNM2*	23974872	Schizophrenia	12q24.31	rs11532322-A	intron_variant
(*2*) *PITPNM2*	25056061	Schizophrenia	12q24.31	rs2851447-G	intron_variant
(*3*) *PITPNM2*	28540026	Autism spectrum disorder or schizophrenia	12q24.31	rs2851447-?	intron_variant
**PI binding proteins**					
***PI3P***					
(*1*) *WDFY3*	26252872	Cerebral amyloid deposition (PET imaging)	4q21.23	rs76117213-G	intron_variant
(*2*) *WDFY3*	26252872	Cerebral amyloid deposition (PET imaging)	4q21.23	rs13152543-A	intergenic_variant
(*1*) *NISCH*	23974872	Schizophrenia	3p21.1	rs4687552-T	non-_coding_transcript_exon_variant
(*2*) *NISCH*	25056061	Schizophrenia	3p21.1	rs2535627-T	downstream_gene_variant
(*3*) *NISCH*	21926972	Bipolar disorder	3p21.1	rs736408-C	intron_variant
(*4*) *NISCH*	28540026	Autism spectrum disorder or schizophrenia	3p21.1	rs3617-?	missense_variant
(*5*) *NISCH*	28540026	Autism spectrum disorder or schizophrenia	3p21.2	rs353547-?	intron_variant
*MTMR4*	25644384	Cognitive function	17q22	rs2429369-?	intron_variant
*ANKFY1*	24039173	Functional impairment in major depressive disorder, bipolar disorder and schizophrenia	17p13.2	rs7221595-?	intron_variant
***PI4P***					
*GGA1*	22041458	Response to anti-depressant treatment in major depressive disorder	22q13.1	rs12157904-G	upstream_gene_variant
*PLEKHA3*	26746183	Rapid functional decline in sporadic amyotrophic lateral sclerosis		chr2:179179368916-C	
***PI5P***					
*UHRF1*	26198764	Schizophrenia	19p13.3	rs34232444-T	upstream_gene_variant
***PI(4,5)P_2_***					
*AP2M1*	22472876	Major depressive disorder	3q27.1	rs1969253-?	intron_variant
(*1*) *SNAP91*	28540026	Autism spectrum disorder or schizophrenia	6q14.2	rs7752643-C	intron_variant
(*2*) *SNAP91*	26198764	Schizophrenia	6q14.2	rs3798869-G	intron_variant
(*3*) *SNAP91*	23092984	Bipolar disorder with mood-incongruent psychosis	6q14.2	rs1171113-C	intron_variant
(*1*) *SH3GL2*	19734545	Cognitive performance	9p22.2	rs10810865-?	intergenic_variant
(*2*) *SH3GL2*	22451204	Parkinson’s disease	9p22.2	rs1536076-?	intron_variant
(*3*) *SH3GL2*	27182965	Parkinson’s disease	9p22.2	rs2209440-?	intron_variant
(*4*) *SH3GL2*	19734545	Cognitive performance	9p22.2	rs4284125-?	intergenic_variant
(*5*) *SH3GL2*	27846195	Response to paliperidone in schizophrenia (negative Marder score)	9p22.2	rs141473550-A	intergenic_variant
*EPN1*	23377640	Major depressive disorder	19q13.42	rs17634917-G	upstream_gene_variant
*RDX*	24684796	Cognitive function	11q22.3	rs7945071-T	intron_variant
(*1*) *KCNJ2*	22648509	Formal thought disorder in schizophrenia	17q24.3	rs1015657-?	intergenic_variant
(*2*) *KCNJ2*	26297903	Depressive episodes in bipolar disorder	17q24.3	rs2190547-?	intergenic_variant
*KCNJ6*	22554406	Electroencephalographic traits in alcoholism	21q22.13	rs2835872-G	intron_variant
*KCNJ11*	24564958	Social communication problems	11p15.1	rs1557765-C	non-_coding_transcript_exon_variant
*CAPN2*	26198764	Schizophrenia	1q41	rs7539624-A	intron_variant
*MTSS1*	24684796	Cognitive function	8q24.13	rs2116081-T	intron_variant
*GSN*	20889312	Bipolar disorder and schizophrenia	9q33.2	rs767770-?	downstream_gene_variant
*VIL1*	22959728	Amyotrophic lateral sclerosis	2q35	rs7607369-A	upstream_gene_variant
*SOS1*	26077951	Corticobasal degeneration	2p22.1	rs963731-?	intron_variant
*TIAM1*	20801718	Amyotrophic lateral sclerosis	21q22.11	rs13048019-T	intron_variant
(*1*) *SPTBN2*	28115744	Bipolar disorder	11q13.2	rs10896135-G	intron_variant
(*2*) *SPTBN2*	21926972	Bipolar disorder	11q13.2	rs10896135-G	intron_variant
(*1*) *GAP43*	28632202	Borderline personality disorder	3q13.31	rs283386-G	intron_variant
(*2*) *GAP43*	26989097	Response to cognitive-behavioral therapy in anxiety disorder	3q13.31	rs16823934-?	intergenic_variant
(*1*) *TRPM8*	24529757	Amyotrophic lateral sclerosis (sporadic)	2q37.1	rs1987842-?	downstream_gene_variant
(*2*) *TRPM8*	27322543	Migraine without aura	2q37.1	rs6724624-?	intergenic_variant
(*3*) *TRPM8*	27182965	Migraine	2q37.1	rs1965629-?	upstream_gene_variant
(*4*) *TRPM8*	22683712	Migraine	2q37.1	rs10166942-?	upstream_gene_variant
(*5*) *TRPM8*	21666692	Migraine	2q37.1	rs10166942-T	upstream_gene_variant
(*6*) *TRPM8*	23793025	Migraine	2q37.1	rs6741751-?	intron_variant
(*7*) *TRPM8*	27322543	Migraine	2q37.1	rs10166942-?	upstream_gene_variant
***PI(3,4,5)P_3_***					
*CYTH1*	24047446	Anxiety and major depressive disorder	17q25.3	rs4796827-A	intergenic_variant
*PHLDB2*	20125193	Cognitive performance	3q13.2	rs4450776-?	intron_variant
*MYO10*	23377640	Major depressive disorder	5p15.1	rs17651119-C	intron_variant
***PI(3,4) P_2_***					
*PLEKHA2*	25993607	Neuroticism	8p11.22	rs11782824-A	intron_variant
***PI(3,5)P_2_***					
(*1*)*CLVS1*	24529757	Amyotrophic lateral sclerosis (sporadic)	8q12.2	rs7830371-?	intron_variant
(*2*) *CLVS1*	23793025	Migraine	8q12.2	rs12681792-?	intron_variant
*RPTOR*	26198764	Schizophrenia	17q25.3	rs8066384-C	intron_variant
**PI binding proteins with degenerate specificity**					
***PI3P, PI4P, PI5P***					
*SEC23IP*	26545630	Cerebrospinal fluid clusterin levels	10q26.12	rs2456721-?	intron_variant
*MTMR3*	28247064	Cerebrospinal P-tau181p levels	22q12.2	rs41157-T	non-_coding_transcript_exon_variant
***PI3P, PI4P***					
(*1*) *FRMD6*	20171287	Brain structure	14q22.1	rs7140150-?	intron_variant
(*2*) *FRMD6*	26252872	Cognitive decline rate in late mild cognitive impairment	14q22.1	rs192549394-G	intron_variant
***PI3P, PI(4,5)P_2_***					
*NUMB*	23092984	Bipolar disorder with mood-incongruent psychosis	14q24.3	rs2333194-?	intron_variant
***PI(4,5)P_2_, PI(3,4,5)P_3_***					
*SEPT5*	26830138	Alzheimer disease and age of onset	22q11.21	rs141503849-T	intergenic_variant
***PI(3,4)P_2_, PI(3,4,5)P_3_***					
*DAPP1*	20195266	Response to antipsychotic treatment	4q23	rs11735070-?	intergenic_variant
*ARAP3*	26252872	Cerebral amyloid deposition (PET imaging)	5q31.3	rs57450513-C	regulatory_region_variant
*AKT2*	26252872	Cerebrospinal T-tau levels	19q13.2	rs76137255-T	intron_variant
(*1*) *AKT3*	21441570	Diabetic retinopathy	1q44	rs476141-A	intron_variant
(*2*) *AKT3*	21441570	Diabetic retinopathy	1q44	rs10927101-A	intron_variant
(*3*) *AKT3*	28346443	Non-glioblastoma glioma	1q44	rs12076373-G	intron_variant
(*4*) *AKT3*	23726511	Post-traumatic stress disorder (adjusted for relatedness)	1q44	rs4430311-?	upstream_gene_variant
(*5*) *AKT3*	23974872	Schizophrenia	1q44	rs14403-C	3_prime_UTR_variant
(*6*) *AKT3*	26198764	Schizophrenia	1q44	rs13376709-C	intron_variant
(*7*) *AKT3*	25056061	Schizophrenia	1q43	rs77149735-A	intron_variant
(*1*) *FERMT2*	27064256	Glaucoma (primary angle closure)	14q22.1	rs7494379-G	intron_variant
(*2*) *FERMT2*	24162737	Alzheimer’s disease (late onset)	14q22.1	rs17125944-C	intron_variant
***PI3P, PI4P, PI(3,4)P_2_, PI(3,5)P_2_, PI(4,5)P_2_***					
*SGIP1*	28641921	Cerebrospinal fluid t-tau:AB1-42 ratio	1p31.3	rs6662771-?	intron_variant
***PI(3,4)P_2_, PI(4,5)P_2_, PI(3,5)P_2_, PI(3,4,5)P_3_***					
(*1*) *SNX9*	28632202	Borderline personality disorder	6q25.3	rs6922614-T	intron_variant
(*2*) *SNX9*	26830138	Alzheimer disease and age of onset	6q25.3	rs34804891-T	intron_variant
***PI(3,4,5)P_3_, PI(3,4)P_2_, PI(4,5)P_2_***					
*TIAM1*	20801718	Amyotrophic lateral sclerosis	21q22.11	rs13048019-T	intron_variant

*The table reports the diseases linked to genes (phosphoinositide signaling pathway genes) based on GWAS studies. For every gene the disease trait, affected chromosomal region, risk allele and type of variant are reported. The rs ID represents the dbSNP ID for each gene.*

## Conclusion

In the past few years, our understanding of phosphoinositide function in cell biology and physiology has rapidly advanced. Although these are quantitatively minor lipids, their impact on neuronal cell biology and function is clearly widespread. Most of these studies were done in experimentally tractable model organisms with a complex nervous system such as *Drosophila*, *Caenorhabditis elegans* or rodents and indicate a crucial role of phosphoinositides in orchestrating major subcellular pathways. Over the same period, advances in next generation DNA sequencing technology and related techniques in human genetic analysis have thrown up a large number of DNA sequence variants and suggested links between these variants and human diseases (Guerreiro et al., 2014; Splinter et al., 2018; Ganapathy et al., 2019); such observations are also true for genes involved in phosphoinositide signaling and their potential function in the context of diseases of the human nervous system. However, there have been two major challenges in this area of science (i) the ability to observe and measure aspects of the cell biology of human brain cells and to test the significance of the observations experimentally (ii) evaluating the functional significance of genetic variants reported in the context of brain disorders in genes related to phosphoinositide signaling. Recent technological advances offer the promise of rapid advances and progress in this area. The difficulty in obtaining live biopsy samples of the human brain has been a major deterrent in studying cellular mechanisms underlying neurological disorders. One possible solution to the inability to observe and experimentally manipulate human brain cells has emerged over the last 10 years in the form of induced pluripotent stem (iPS) cell technology. Using this approach one can derive pluripotent stem cells from somatic human tissue through reprograming (Shi et al., 2017). These reprogrammed stem cells can then be differentiated into neural cell types and used to study neural cell biology and physiology.

To date, several neurological diseases have been modeled based on patient-derived iPS cell technology and subsequent neural differentiation to study cellular mechanisms in disease (Russo et al., 2015; Wen et al., 2016). A recent study by Mertens et al., using iPSC technology, has revealed mitochondrial abnormalities and neuronal hyper-excitability in young neurons derived from BD patients; this hyperexcitability can be reversed by Li^+^ treatment only in neurons derived from Li^+^ sensitive patients (Mertens et al., 2015). Another recent study on iPSC derived neurons from Parkinson’s disease patients has revealed defects in the regulation of ER Ca^2+^ stores, which can be linked to the PI(4,5)P_2_ cycle since it actively regulates Ca^2+^ homeostasis (Korecka et al., 2019). An iPSC model for Lowe Syndrome has also been developed by Barnes et al., that suggests abnormalities in F-actin polymerization, WAVE-1 expression and altered PI(4,5)P_2_ levels in patient specific iPSC derived neurons, giving a further insight about the disease pathology at the cellular level (Barnes et al., 2018).

A second major development in recent times is the development of genome engineering technologies such as Zn^2+^ finger nucleases, TALEN and CRISPR/Cas9 that allow DNA sequence changes to be introduced into many human cell types including iPS cells. By adopting this approach, one can experimentally test the contributions of DNA sequence variants described in patient samples to the development of disease phenotypes, as demonstrated by Barnes et al. (2018). Together, these approaches are likely to accelerate our understanding of the role of phosphoinositide signaling in human disease biology in relation to the nervous system.

## Author Contributions

PR conceptualized and wrote the review. AJ, HK, PS, and SS wrote the review. HK and AJ mined public databases and collated gene expression data and disease data.

## Conflict of Interest Statement

The authors declare that the research was conducted in the absence of any commercial or financial relationships that could be construed as a potential conflict of interest.

## Footnotes

1 http://www.brainrnaseq.org

2 https://gtexportal.org/home/

3 https://www.libd.org/brain-cloud/

4 https://www.omim.org

5 http://www.informatics.jax.org

6 http://www.ebi.ac.uk/gwas

## References

[B1] Agam G. Bersudsky Y. Berry G. T. Moechars D. Lavi-Avnon Y. Belmaker R. H. (2009). Knockout mice in understanding the mechanism of action of lithium. *Biochem. Soc. Trans.* 37 1121–1125. 10.1042/BST0371121 19754464

[B2] Al Zaabi N. Noora Z. Al-Jasmi F. (2018). SYNJ1 gene associated with neonatal onset of neurodegenerative disorder and intractable seizure. *Mol. Genet. Genomic Med.* 6 109–113. 10.1002/mgg3.341 29179256 PMC5823681

[B3] Alcantara D. Timms A. E. Gripp K. Baker L. Park K. Collins S. (2017). Mutations of AKT3 are associated with a wide spectrum of developmental disorders including extreme megalencephaly. *Brain* 140 2610–2622. 10.1093/brain/awx203 28969385 PMC6080423

[B4] Al-Ramahi I. Giridharan S. S. P. Chen Y.-C. Patnaik S. Safren N. Hasegawa J. (2017). Inhibition of PIP4Kg ameliorates the pathological effects of mutant huntingtin protein. *eLife* 6:e29123. 10.7554/eLife.29123 29256861 PMC5743427

[B5] Arai Y. Ijuin T. Takenawa T. Becker L. E. Takashima S. (2002). Excessive expression of synaptojanin in brains with Down syndrome. *Brain Dev.* 24 67–72. 10.1016/S0387-7604(01)00405-3 11891094

[B6] Arendt K. L. Benoist M. Lario A. Draffin J. E. Munoz M. Esteban J. A. (2014). PTEN counteracts PIP3 upregulation in spines during NMDA-receptor-dependent long-term depression. *J. Cell Sci.* 127 5253–5260. 10.1242/jcs.156554 25335889

[B7] Azzedine H. Bolino A. Taïeb T. Birouk N. Di Duca M. Bouhouche A. (2003). Mutations in MTMR13, a new pseudophosphatase homologue of MTMR2 and Sbf1, in two families with an autosomal recessive demyelinating form of charcot-marie-tooth disease associated with early-onset glaucoma. *Am. J. Hum. Genet.* 72 1141–1153. 10.1086/375034 12687498 PMC1180267

[B8] Bae B.-I. Jayaraman D. Walsh C. A. (2015). Genetic changes shaping the human brain. *Dev. Cell* 32 423–434. 10.1016/j.devcel.2015.01.035 25710529 PMC4429600

[B9] Bakkour A. Morris J. C. Wolk D. A. Dickerson B. C. (2013). The effects of aging and Alzheimer’s disease on cerebral cortical anatomy: specificity and differential relationships with cognition. *Neuroimage* 76 332–344. 10.1016/j.neuroimage.2013.02.059.The 23507382 PMC4098706

[B10] Balakrishnan S. S. Basu U. Raghu P. (2015). Phosphoinositide signalling in *Drosophila*. *Biochim. Biophys. Acta Mol. Cell Biol. Lipids* 1851 770–784. 10.1016/j.bbalip.2014.10.010 25449646

[B11] Balakrishnan S. S. Basu U. Shinde D. Thakur R. Jaiswal M. Raghu P. (2018). Regulation of PI4P levels by PI4KIIIα during G-protein-coupled PLC signaling in *Drosophila* photoreceptors. *J. Cell Sci.* 131:jcs217257. 10.1242/jcs.217257 29980590 PMC6104824

[B12] Balla A. Balla T. (2006). Phosphatidylinositol 4-kinases: old enzymes with emerging functions. *Trends Cell Biol.* 16 351–361. 10.1016/j.tcb.2006.05.003 16793271

[B13] Balla T. (2005). Inositol-lipid binding motifs: signal integrators through protein-lipid and protein-protein interactions. *J. Cell. Sci.* 118 2093–2104. 10.1242/jcs.02387 15890985

[B14] Balla T. (2013). Phosphoinositides: tiny lipids with giant impact on cell regulation. *Physiol. Rev.* 93 1019–1137. 10.1152/physrev.00028.2012 23899561 PMC3962547

[B15] Barnes J. Salas F. Mokhtari R. Dolstra H. Pedrosa E. Lachman H. M. (2018). Modeling the neuropsychiatric manifestations of Lowe syndrome using induced pluripotent stem cells: defective F-actin polymerization and WAVE-1 expression in neuronal cells. *Mol. Autism* 9 1–16. 10.1186/s13229-018-0227-3 30147856 PMC6094927

[B16] Baulac S. Lenk G. M. Couarch P. Larson P. A. Ferguson C. J. Noé E. (2014). Role of the phosphoinositide phosphatase FIG4 gene in familial epilepsy with polymicrogyria. *Neurology* 82 1068–1075. 10.1212/WNL.0000000000000241 24598713 PMC3962989

[B17] Berridge M. J. Downes C. P. Hanley M. R. (1989). Neural and developmental actions of lithium: a unifying hypothesis. *Cell* 59 411–419. 10.1016/0092-8674(89)90026-3 2553271

[B18] Berridge M. J. Downest C. P. Hanleyt M. R. (1982). Lithium amplifies agonist-dependent phosphatidylinositol responses in brain and salivary glands. *Biochem. J.* 206 587–595. 10.1042/bj2060587 7150264 PMC1158627

[B19] Berridge M. J. Irvine R. F. (1984). Inositol trisphosphate, a novel second messenger in cellular signal transduction. *Nature* 312 315–321. 10.1038/312315a0 6095092

[B20] Berridge M. J. Irvine R. F. (1989). Inositol phosphates and cell signalling. *Nature* 341 197–205. 10.1038/341197a0 2550825

[B21] Bielas S. L. Silhavy J. L. Brancati F. Kisseleva M. V. Al-gazali L. Sztriha L. (2009). Mutations in INPP5E, encoding inositol polyphosphate- 5-phosphatase E, link phosphatidyl inositol signaling to the ciliopathies. *Nat. Genet.* 41 1032–1036. 10.1038/ng.423 19668216 PMC2746682

[B22] Boal F. Mansour R. Gayral M. Saland E. Chicanne G. Xuereb J.-M. (2015). TOM1 is a PI5P effector involved in the regulation of endosomal maturation. *J. Cell Sci.* 128 815–827. 10.1242/jcs.166314 25588840

[B23] Bökenkamp A. Ludwig M. (2016). The oculocerebrorenal syndrome of Lowe: an update. *Pediatr. Nephrol.* 31 2201–2212. 10.1007/s00467-016-3343-3 27011217 PMC5118406

[B24] Bolino A. Muglia M. Conforti F. L. LeGuern E. Salih M. A. M. Georgiou D.-M. (2000). Charcot-Marie-Tooth type 4B is caused by mutations in the gene encoding myotubularin-related protein-2. *Nat. Genet.* 25 17–19. 10.1038/75542 10802647

[B25] Butler M. G. Dasouki M. J. Zhou X.-P. Talebizadeh Z. Brown M. Takahashi T. N. (2005). Subset of individuals with autism spectrum disorders and extreme macrocephaly associated with germline PTEN tumour suppressor gene mutations. *J. Med. Genet.* 42 318–321. 10.1136/jmg.2004.024646 15805158 PMC1736032

[B26] Cade J. F. (1949). Lithium salts in the treatment of psychotic excitement. *Med. J. Aust.* 2 349–352. 10.3109/00048678209159969 18142718

[B27] Campeau P. M. Lenk G. M. Lu J. T. Bae Y. Burrage L. Morandi L. (2013). Yunis-Varon syndrome is caused by mutations in FIG4, encoding a phosphoinositide phosphatase. *Am. J. Hum. Genet.* 92 781–791. 10.1016/j.ajhg.2013.03.020 23623387 PMC3644641

[B28] Chakrabarti P. Kolay S. Yadav S. Kumari K. Nair A. Trivedi D. (2015). A dPIP5K dependent pool of phosphatidylinositol 4,5 bisphosphate (PIP2) is required for G-protein coupled signal transduction in *Drosophila* photoreceptors. *PLoS Genet.* 11:e1004948. 10.1371/journal.pgen.1004948 25633995 PMC4310717

[B29] Chow C. Y. Landers J. E. Bergren S. K. Sapp P. C. Grant A. E. Jones J. M. (2009). Deleterious variants of FIG4, a phosphoinositide phosphatase, in patients with ALS. *Am. J. Hum. Genet.* 84 85–88. 10.1016/j.ajhg.2008.12.010 19118816 PMC2668033

[B30] Chow C. Y. Zhang Y. Dowling J. J. Jin N. Adamska M. Shiga K. (2007). Mutation of FIG4 causes neurodegeneration in the pale tremor mouse and patients with CMT4J. *Nature* 448 68–72. 10.1038/nature05876 17572665 PMC2271033

[B31] Cockcroft S. Raghu P. (2018). Phospholipid transport protein function at organelle contact sites. *Curr. Opin. Cell Biol.* 53 52–60. 10.1016/j.ceb.2018.04.011 29859321 PMC6141807

[B32] Colantuoni C. Lipska B. K. Ye T. Hyde T. M. Tao R. Leek J. T. (2011). Temporal dynamics and genetic control of transcription in the human prefrontal cortex. *Nature* 478 519–523. 10.1038/nature10524 22031444 PMC3510670

[B33] Cossec J.-C. Lavaur J. Berman D. E. Rivals I. Hoischen A. Stora S. (2012). Trisomy for Synaptojanin1 in Down syndrome is functionally linked to the enlargement of early endosomes. *Hum. Mol. Genet.* 21 3156–3172. 10.1093/hmg/dds142 22511594 PMC3384382

[B34] Cremona O. Di Paolo G. Wenk M. R. Lüthi A. Kim W. T. Takei K. (1999). Essential role of phosphoinositide metabolism in synaptic vesicle recycling. *Cell* 99 179–188. 10.1016/s0092-8674(00)81649-9 10535736

[B35] D’Angelo G. Vicinanza M. Di Campli A. De Matteis M. A. (2008). The multiple roles of PtdIns(4)P – not just the precursor of PtdIns(4,5)P2. *J. Cell Sci.* 121 1955–1963. 10.1242/jcs.023630 18525025

[B36] Darmanis S. Sloan S. A. Zhang Y. Enge M. Caneda C. Shuer L. M. (2015). A survey of human brain transcriptome diversity at the single cell level. *Proc. Natl. Acad. Sci. U.S.A.* 112 7285–7290. 10.1073/pnas.1507125112 26060301 PMC4466750

[B37] Dawson R. M. (1954a). Studies on the labelling of brain phospholipids with radioactive phosphorus. *Biochem. J.* 57 237–245. 10.1042/bj0570237 13172175 PMC1269738

[B38] Dawson R. M. (1954b). The measurement of 32P labelling of individual kephalins and lecithin in a small sample of tissue. *Biochim. Biophys. Acta* 14 374–379. 10.1016/0006-3002(54)90195-x 13181893

[B39] De Paula M. L. Cui Q.-L. Hossain S. Antel J. Almazan G. (2014). The PTEN inhibitor bisperoxovanadium enhances myelination by amplifying IGF-1 signaling in rat and human oligodendrocyte progenitors.pdf. *Glia* 62 64–77. 10.1002/glia.22584 24166839

[B40] Dennis E. A. (2015). Introduction to thematic review series: phospholipases: central role in lipid signaling and disease. *J. Lipid Res.* 56 1245–1247. 10.1194/jlr.E061101 26031662 PMC4479329

[B41] Di Paolo G. De Camilli P. (2006). Phosphoinositides in cell regulation and membrane dynamics. *Nature* 443 651–657. 10.1038/nature05185 17035995

[B42] Di Paolo G. Moskowitz H. S. Gipson K. Wenk M. R. Voronov S. Obayashi M. (2004). Impaired PtdIns (4,5)P2 synthesis in nerve terminals produces defects in synaptic vesicle trafficking. *Nature* 431 415–422. 10.1038/nature02896 15386003

[B43] Dickson E. J. Hille B. (2019). Understanding phosphoinositides: rare, dynamic, and essential membrane phospholipids. *Biochem. J.* 476 1–23. 10.1042/BCJ20180022 30617162 PMC6342281

[B44] Dickson E. J. Jensen J. B. Hille B. (2014). Golgi and plasma membrane pools of PI(4)P contribute to plasma membrane PI(4,5)P2 and maintenance of KCNQ2/3 ion channel current. *Proc. Natl. Acad. Sci. U.S.A.* 111 E2281–E2290. 10.1073/pnas.1407133111 24843134 PMC4050574

[B45] Divecha N. Banfić H. Irvine R. F. (1993). Inositides and the nucleus and inositides in the nucleus. *Cell* 74 405–407. 10.1016/0092-8674(93)80041-c 8394217

[B46] Donaghy M. (ed) (2011). *Brain’s Diseases of the Nervous System*, 12th Edn Oxford: Oxford University Press, 10.1093/med/9780198569381.001.0001

[B47] Dyment D. A. Smith A. C. Humphreys P. Schwartzentruber J. Beaulieu C. L. Consortium F. C. (2015). Neurobiology of Aging Homozygous nonsense mutation in SYNJ1 associated with intractable epilepsy and tau pathology. *Neurobiol. Aging* 36:1222.e1-.e5. 10.1016/j.neurobiolaging.2014.09.005 25316601

[B48] Engelman J. A. Luo J. Cantley L. C. (2006). The evolution of phosphatidylinositol 3-kinases as regulators of growth and metabolism. *Nat. Rev. Genet.* 7 606–619. 10.1038/nrg1879 16847462

[B49] Fedorenko O. Y. Loonen A. J. M. Lang F. Toshchakova V. A. Boyarko E. G. Semke A. V. (2015). Association study indicates a protective role of phosphatidylinositol-4-phosphate-5-kinase against Tardive Dyskinesia. *Int. J. Neuropsychopharmacol.* 18:pyu098. 10.1093/ijnp/pyu098 25548108 PMC4438543

[B50] Fiume R. Stijf-Bultsma Y. Shah Z. H. Keune W. J. Jones D. R. Jude J. G. (2015). PIP4K and the role of nuclear phosphoinositides in tumour suppression. *Biochim. Biophys. Acta Mol. Cell Biol. Lipids* 1851 898–910. 10.1016/j.bbalip.2015.02.014 25728392

[B51] Folch J. (1949a). Brain diphosphoninositide, a new phosphatide having inositol metadiphosphate as a constituent. *J. Biol. Chem.* 177 505–519. 18110428

[B52] Folch J. (1949b). Complete fractionation of brain cephalin; isolation from it of phosphatidyl serine, phosphatidyl ethanolamine, and diphosphoinositide. *J. Biol. Chem.* 177 497–504. 18110427

[B53] Folch J. Wolleey D. W. (1942). Inositol, a constituent of brain phosphatidate. *J. Biol. Chem* 142 963–964.

[B54] Franz H. Ullmann C. Becker A. Ryan M. Bahn S. Arendt T. (2005). Systematic analysis of gene expression in human brains before and after death. *Genome Biol.* 6:R112. 10.1186/gb-2005-6-13-r112 16420671 PMC1414111

[B55] Frazier T. W. Embacher R. Tilot A. K. Koenig K. Mester J. Eng C. (2015). Molecular and phenotypic abnormalities in individuals with germline heterozygous PTEN mutations and autism. *Mol. Psychiatry* 20 1132–1138. 10.1038/mp.2014.125 25288137 PMC4388743

[B56] Freland L. Beaulieu J.-M. (2012). Inhibition of GSK3 by lithium, from single molecules to signaling networks. *Front. Mol. Neurosci.* 5:14. 10.3389/fnmol.2012.00014 22363263 PMC3282483

[B57] Friedman E. Hoau-Yan-Wang, Levinson D. Connell T. A. Singh H. (1993). Altered platelet protein kinase C activity in bipolar affective disorder, manic episode. *Biol. Psychiatry* 33 520–525. 10.1016/0006-3223(93)90006-Y 8513036

[B58] Funakoshi Y. Hasegawa H. Kanaho Y. (2011). Regulation of PIP5K activity by Arf6 and its physiological significance. *J. Cell. Physiol.* 226 888–895. 10.1002/jcp.22482 20945365

[B59] Gaidarov I. Smith M. E. Domin J. Keen J. H. (2001). The class II phosphoinositide 3-kinase C2alpha is activated by clathrin and regulates clathrin-mediated membrane trafficking. *Mol. Cell* 7 443–449. 10.1016/s1097-2765(01)00191-5 11239472

[B60] Ganapathy A. Mishra A. Soni M. R. Kumar P. Sadagopan M. Kanthi A. V. (2019). Multi-gene testing in neurological disorders showed an improved diagnostic yield: data from over 1000 Indian patients. *J. Neurol.* 266 1919–1926. 10.1007/s00415-019-09358-1 31069529

[B61] Gehler S. Shaw A. E. Sarmiere P. D. Bamburg J. R. Letourneau P. C. (2004). Brain-derived neurotrophic factor regulation of retinal growth cone filopodial dynamics is mediated through actin depolymerizing factor/cofilin. *J. Neurosci.* 24 10741–10749. 10.1523/JNEUROSCI.2836-04.2004 15564592 PMC6730129

[B62] Godi A. Pertile P. Meyers R. Marra P. Di Tullio G. Iurisci C. (1999). ARF mediates recruitment of PtdIns-4-OH kinase-beta and stimulates synthesis of PtdIns(4,5)P2 on the Golgi complex. *Nat. Cell Biol.* 1 280–287. 10.1038/12993 10559940

[B63] Goebbels S. Wieser G. L. Pieper A. Spitzer S. Weege B. Yan K. (2017). A neuronal PI(3,4,5)P3-dependent program of oligodendrocyte precursor recruitment and myelination. *Nat. Neurosci.* 20 10–15. 10.1038/nn.4425 27775720

[B64] Goes F. S. Mcgrath J. Avramopoulos D. Wolyniec P. Pirooznia M. Ruczinski I. (2015). Genome-wide association study of schizophrenia in Ashkenazi Jews. *Am. J. Med. Genet. Part B Neuropsychiatr. Genet.* 168 649–659. 10.1002/ajmg.b.32349 26198764

[B65] Gong L.-W. De Camilli P. (2008). Regulation of postsynaptic AMPA responses by synaptojanin 1. *Proc. Natl. Acad. Sci. U.S.A.* 105 17561–17566. 10.1073/pnas.0809221105 18987319 PMC2579885

[B66] Grimm J. Sachs M. Britsch S. Di Cesare S. Schwarz-Romond T. Alitalo K. (2001). Novel p62dok family members, dok-4 and dok-5, are substrates of the c-Ret receptor tyrosine kinase and mediate neuronal differentiation. *J. Cell Biol.* 154 345–354. 10.1083/jcb.200102032 11470823 PMC2150770

[B67] Guerreiro R. Bras J. Hardy J. Singleton A. (2014). Next generation sequencing techniques in neurological diseases: redefining clinical and molecular associations. *Hum. Mol. Genet.* 23 R47–R53. 10.1093/hmg/ddu203 24794858 PMC4170717

[B68] Guo J. Wenk M. R. Pellegrini L. Onofri F. Benfenati F. De Camilli P. (2003). Phosphatidylinositol 4-kinase type IIalpha is responsible for the phosphatidylinositol 4-kinase activity associated with synaptic vesicles. *Proc. Natl. Acad. Sci. U.S.A.* 100 3995–4000. 10.1073/pnas.0230488100 12646710 PMC153036

[B69] Gupta A. Toscano S. Trivedi D. Jones D. R. Mathre S. Clarke J. H. (2013). Phosphatidylinositol 5-phosphate 4-kinase (PIP4K) regulates TOR signaling and cell growth during *Drosophila* development. *Proc. Natl. Acad. Sci. U.S.A.* 110 5963–5968. 10.1073/pnas.1219333110 23530222 PMC3625332

[B70] Hagenauer M. H. Schulmann A. Li J. Z. Vawter M. P. Walsh D. M. Thompson R. C. (2018). Inference of cell type content from human brain transcriptomic datasets illuminates the effects of age, manner of death, dissection, and psychiatric diagnosis. *PLoS One* 13:e0200003. 10.1371/journal.pone.0200003 30016334 PMC6049916

[B71] Hallcher L. M. Sherman W. R. (1980). The effects of lithium ion and other agents on the activity of myo- inositol-1-phosphatase from bovine brain. *J. Biol. Chem.* 255 10896–10901. 6253491

[B72] Hammond G. R. V. Balla T. (2015). Polyphosphoinositide binding domains: key to inositol lipid biology. *Biochim. Biophys. Acta* 1851 746–758. 10.1016/j.bbalip.2015.02.013 25732852 PMC4380703

[B73] Hampshire D. J. Ayub M. Springell K. Roberts E. Jafri H. Rashid Y. (2006). MORM syndrome (mental retardation, truncal obesity, retinal dystrophy and micropenis), a new autosomal recessive disorder, links to 9q34. *Eur. J. Hum. Genet.* 14 543–548. 10.1038/sj.ejhg.5201577 16493448

[B74] Hansen S. B. (2015). Lipid agonism: the PIP2 paradigm of ligand-gated ion channels. *Biochim. Biophys. Acta Mol. Cell Biol. Lipids* 1851 620–628. 10.1016/j.bbalip.2015.01.011 25633344 PMC4540326

[B75] Hardies K. Cai Y. Jardel C. Jansen A. C. Cao M. May P. (2016). Loss of SYNJ1 dual phosphatase activity leads to early onset refractory seizures and progressive neurological decline. *Brain* 139(Pt 9), 2420–2430. 10.1093/brain/aww180 27435091 PMC4995362

[B76] Harrington E. P. Zhao C. Fancy S. P. J. Franklin R. J. M. Rowitch D. H. (2010). Oligodendrocyte PTEN is required for myelin and axonal integrity, not remyelination. *Ann. Neurol.* 68 703–716. 10.1002/ana.22090.Oligodendrocyte 20853437 PMC2966537

[B77] Harwood A. J. (2005). Lithium and bipolar mood disorder: the inositol-depletion hypothesis revisited. *Mol. Psychiatry* 10 117–126. 10.1038/sj.mp.4001618 15558078

[B78] Hasegawa J. Strunk B. S. Weisman L. S. (2017). PI5P and PI(3,5)P2: minor, but essential phosphoinositides. *Cell Struct. Funct.* 42 49–60. 10.1247/csf.17003 28302928 PMC5846621

[B79] Haucke V. (2005). Phosphoinositide regulation of clathrin-mediated endocytosis. *Biochem. Soc. Trans.* 33 1285–1289. 10.1042/BST20051285 16246100

[B80] Hawkins P. T. Stephens L. R. (2016). Emerging evidence of signalling roles for PI(3,4)P2 in Class I and II PI3K-regulated pathways. *Biochem. Soc. Trans.* 44 307–314. 10.1042/BST20150248 26862220

[B81] He K. Marsland R. III Upadhyayula S. Song E. Dang S. Capraro B. R. (2017). Dynamics of phosphoinositide conversion in clathrin-mediated endocytic traffic. *Nature* 552 410–414. 10.1038/nature25146 29236694 PMC6263037

[B82] Henle S. J. Wang G. Liang E. Wu M. Poo M. Henley J. R. (2011). Asymmetric PI(3,4,5)P3 and Akt signaling mediates chemotaxis of axonal growth cones. *J. Neurosci.* 31 7016–7027. 10.1523/JNEUROSCI.0216-11.2011 21562263 PMC3133771

[B83] Herold C. Hooli B. V. Mullin K. Liu T. Roehr J. T. Mattheisen M. (2016). Family-based association analyses of imputed genotypes reveal genome-wide significant association of Alzheimer’s disease with OSBPL6, PTPRG, and PDCL3. *Mol. Psychiatry* 21 1608–1612. 10.1038/mp.2015.218 26830138 PMC4970971

[B84] Hill E. van Der Kaay J. Downes C. P. Smythe E. (2001). The role of dynamin and its binding partners in coated pit invagination and scission. *J. Cell Biol.* 152 309–323. 11266448 10.1083/jcb.152.2.309 PMC2199618

[B85] Hille B. Dickson E. J. Kruse M. Vivas O. Suh B.-C. (2015). Phosphoinositides regulate ion channels. *Biochim. Biophys. Acta Mol. Cell Biol. Lipids* 1851 844–856. 10.1016/j.bbalip.2014.09.010 25241941 PMC4364932

[B86] Hirst J. Motley A. Harasaki K. Peak Chew S. Y. Robinson M. S. (2003). EpsinR: an ENTH domain-containing protein that interacts with AP-1. *Mol. Biol. Cell* 14 625–641. 10.1091/mbc.E02-09-0552 12589059 PMC149997

[B87] Hokin L. E. Hokin M. R. (1955). Effects of acetylcholine on the turnover of phosphoryl units in individual phospholipids of pancreas slices and brain cortex slices. *Biochim. Biophys. Acta* 18 102–110. 10.1016/0006-3002(55)90013-5 13260248

[B88] Hokin M. R. Hokin L. E. Shelp W. D. (1960). The effects of acetylcholine on the turnover of phosphatidic acid and phosphoinositide in sympathetic ganglia, and in various parts of the central nervous system in vitro. *J. Gen. Physiol.* 44 217–226. 10.1085/jgp.44.2.217 13715211 PMC2195095

[B89] Holahan M. R. (2017). A shift from a pivotal to supporting role for the growth-associated protein (GAP-43) in the coordination of axonal structural and functional plasticity. *Front. Cell. Neurosci.* 11:266. 10.3389/fncel.2017.00266 28912688 PMC5583208

[B90] Houlihan L. M. Christoforou A. Arbuckle M. I. Torrance H. S. Anderson S. M. Muir W. J. (2009). A case-control association study and family-based expression analysis of the bipolar disorder candidate gene PI4K2B. *J. Psychiatr. Res.* 43 1272–1277. 10.1016/j.jpsychires.2009.05.004 19539307 PMC2789249

[B91] Howell K. R. Floyd K. Law A. J. (2017). PKBγ/AKT3 loss-of-function causes learning and memory deficits and deregulation of AKT/mTORC2 signaling: relevance for schizophrenia. *PLoS One* 12:e0175993. 10.1371/journal.pone.0175993 28467426 PMC5414975

[B92] Hsuan J. Cockcroft S. (2001). The PITP family of phosphatidylinositol transfer proteins. *Genome Biol.* 2:REVIEWS3011. 10.1186/gb-2001-2-9-reviews3011 11574064 PMC138965

[B93] Ikeda M. Saito T. Kondo K. Iwata N. (2018). Genome-wide association studies of bipolar disorder: a systematic review of recent findings and their clinical implications. *Psychiatry Clin. Neurosci.* 72 52–63. 10.1111/pcn.12611 29057581

[B94] Jansen L. A. Mirzaa G. M. Ishak G. E. Roak B. J. O. Hiatt J. B. Roden W. H. (2015). PI3K/AKT pathway mutations cause a spectrum of brain malformations from megalencephaly to focal cortical dysplasia. *Brain* 138 1613–1628. 10.1093/brain/awv045 25722288 PMC4614119

[B95] Jentsch T. J. (2000). Neuronal KCNQ potassium channels:physislogy and role in disease. *Nat. Rev. Neurosci.* 1 21–30. 10.1038/35036198 11252765

[B96] Jha A. Ahuja M. Patel S. Brailoiu E. Muallem S. (2014). Convergent regulation of the lysosomal two-pore channel-2 by Mg2+, NAADP, PI(3,5)P2 and multiple protein kinases. *EMBO J.* 33 501–511. 10.1002/embj.201387035 24502975 PMC3989630

[B97] Jha M. K. Morrison B. M. (2018). Glia-neuron energy metabolism in health and diseases: new insights into the role of nervous system metabolic transporters. *Exp. Neurol.* 309 23–31. 10.1016/j.expneurol.2018.07.009 30044944 PMC6156776

[B98] Jiang K. Liu Y. Fan J. Zhang J. Li X.-A. Evers B. M. (2016). PI(4)P promotes phosphorylation and conformational change of smoothened through interaction with its C-terminal Tail. *PLoS Biol.* 14:e1002375. 10.1371/journal.pbio.1002375 26863604 PMC4749301

[B99] Jungerius B. J. Hoogendoorn M. L. C. Bakker S. C. Slot R. Van Bardoel A. F. Ophoff R. A. (2008). An association screen of myelin-related genes implicates the chromosome 22q11 PIK4CA gene in schizophrenia. *Mol. Psychiatry* 13 1060–1068. 10.1038/sj.mp.4002080 17893707

[B100] Jungmichel S. Sylvestersen K. B. Choudhary C. Nguyen S. Mann M. Nielsen M. L. (2014). Specificity and commonality of the phosphoinositide-binding proteome analyzed by quantitative mass spectrometry. *Cell Rep.* 6 578–591. 10.1016/j.celrep.2013.12.038 24462288

[B101] Kamalesh K. Trivedi D. Toscano S. Sharma S. Kolay S. Raghu P. (2017). Phosphatidylinositol 5-phosphate 4-kinase regulates early endosomal dynamics during clathrin-mediated endocytosis. *J. Cell Sci.* 130 2119–2133. 10.1242/jcs.202259 28507272 PMC5536888

[B102] Kanellos G. Frame M. C. (2016). Cellular functions of the ADF/cofilin family at a glance. *J. Cell Sci.* 129 3211–3218. 10.1242/jcs.187849 27505888

[B103] Kaur H. Jajodia A. Grover S. Baghel R. Gupta M. Jain S. (2014). Genetic variations of PIP4K2A confer vulnerability to poor antipsychotic response in severely Ill Schizophrenia patients. *PLoS One* 9:e102556. 10.1371/journal.pone.0102556 25025909 PMC4099378

[B104] Kawano M. Kumagai K. Nishijima M. Hanada K. (2006). Efficient trafficking of ceramide from the endoplasmic reticulum to the Golgi apparatus requires a VAMP-associated protein-interacting FFAT motif of CERT. *J. Biol. Chem.* 281 30279–30288. 10.1074/jbc.M605032200 16895911

[B105] Kenworthy L. Park T. Charnas L. R. (1993). Cognitive and behavioral profile of the oculocerebrorenal syndrome of Lowe. *Am. J. Med. Genet.* 46 297–303. 10.1002/ajmg.1320460312 8488875

[B106] Klopfenstein D. R. Tomishige M. Stuurman N. Vale R. D. (2002). Role of phosphatidylinositol(4,5)bisphosphate organization in membrane transport by the Unc104 kinesin motor. *Cell* 109 347–358. 10.1016/s0092-8674(02)00708-0 12015984 PMC2851634

[B107] Klopfenstein D. R. Vale R. D. (2004). The lipid binding pleckstrin homology domain in UNC-104 kinesin is necessary for synaptic vesicle transport in *Caenorhabditis elegans*. *Mol. Biol. Cell* 15 3729–3739. 10.1091/mbc.e04-04-0326 15155810 PMC491832

[B108] Kolay S. Basu U. Raghu P. (2016). Control of diverse subcellular processes by a single multi-functional lipid phosphatidylinositol 4,5-bisphosphate [PI(4,5)P2]. *Biochem. J.* 473 1681–1692. 10.1042/BCJ20160069 27288030 PMC6609453

[B109] Korecka J. A. Talbot S. Osborn T. M. Leeuw S. M. De Levy S. A. Ferrari E. J. (2019). Neurite collapse and altered ER Ca2+ control in human parkinson disease patient iPSC-derived neurons with LRRK2 G2019S mutation. *Stem Cell Rep.* 12 29–41. 10.1016/j.stemcr.2018.11.021 30595548 PMC6335600

[B110] Kovacs G. G. Adle-Biassette H. Milenkovic I. Cipriani S. van Scheppingen J. Aronica E. (2014). Linking pathways in the developing and aging brain with neurodegeneration. *Neuroscience* 269 152–172. 10.1016/j.neuroscience.2014.03.045 24699227

[B111] Krebs C. E. Karkheiran S. Powell J. C. Cao M. Makarov V. Darvish H. (2013). The Sac1 domain of SYNJ1 identified mutated in a family with early-onset progressive Parkinsonism with generalized seizures. *Hum. Mutat.* 34 1200–1207. 10.1002/humu.22372 23804563 PMC3790461

[B112] Kumar J. Choudhary B. C. Metpally R. Zheng Q. Nonet M. L. Ramanathan S. (2010). The *Caenorhabditis elegans* Kinesin-3 motor UNC-104/KIF1A is degraded upon loss of specific binding to cargo. *PLoS Genet.* 6:e1001200. 10.1371/journal.pgen.1001200 21079789 PMC2973836

[B113] Lachyankar M. B. Sultana N. Schonhoff C. M. Mitra P. Poluha W. Lambert S. (2000). A role for nuclear PTEN in neuronal differentiation. *J. Neurosci.* 20 1404–1413. 10.1523/jneurosci.20-04-01404.2000 10662831 PMC6772384

[B114] Lambert J.-C. Ibrahim-Verbaas C. A. Harold D. Naj A. C. Sims R. Bellenguez C. (2013). Meta-analysis of 74, 046 individuals identifies 11 new susceptibility loci for Alzheimer’s disease. *Nat. Genet.* 45 1452–1460. 10.1038/ng.2802 24162737 PMC3896259

[B115] Lenk G. M. Meisler M. H. (2014). Mouse models of PI(3,5)P2 deficiency with impaired lysosome function. *Methods Enzymol.* 534 245–260. 10.1016/B978-0-12-397926-1.00014-17 24359958 PMC4059992

[B116] Logan A. M. Mammel A. E. Robinson D. C. Chin A. L. Condon A. F. Robinson F. L. (2017). Schwann cell-specific deletion of the endosomal PI 3-kinase Vps34 leads to delayed radial sorting of axons, arrested myelination, and abnormal ErbB2-ErbB3 tyrosine kinase signaling. *Glia* 65 1452–1470. 10.1002/glia.23173 28617998 PMC5818149

[B117] Lonsdale J. Thomas J. Salvatore M. Phillips R. Lo E. Shad S. (2013). The genotype-tissue expression (GTEx) project. *Nat. Genet.* 45 580–585. 10.1038/ng.2653 23715323 PMC4010069

[B118] MacArthur J. Bowler E. Cerezo M. Gil L. Hall P. Hastings E. (2017). The new NHGRI-EBI catalog of published genome-wide association studies (GWAS Catalog). *Nucleic Acids Res.* 45 D896–D901. 10.1093/nar/gkw1133 27899670 PMC5210590

[B119] Madsen R. R. Vanhaesebroeck B. Semple R. K. (2018). Cancer-associated PIK3CA mutations in overgrowth disorders. *Trends Mol. Med.* 24 856–870. 10.1016/j.molmed.2018.08.003 30197175 PMC6185869

[B120] Mani M. Lee S. Y. Lucast L. Cremona O. Di Paolo G. De Camilli P. (2007). The dual phosphatase activity of synaptojanin1 is required for both efficient synaptic vesicle endocytosis and reavailability at nerve terminals. *Neuron* 56 1004–1018. 10.1016/j.neuron.2007.10.032 18093523 PMC3653591

[B121] Martin T. F. J. (1998). Phosphoinositide lipids as signalling molecules: common themes for signal transduction, cytoskeletal regulation, and membrane trafficking. *Annu. Rev. Cell Dev. Biol.* 14 231–264. 10.1146/annurev.cellbio.14.1.231 9891784

[B122] Mcbride K. L. Varga E. A. Pastore M. T. Prior T. W. Manickam K. Atkin J. F. (2010). Confirmation study of PTEN mutations among individuals with autism or developmental delays/mental retardation and macrocephaly. *Autism Res.* 3 137–141. 10.1002/aur.132 20533527

[B123] McCartney A. J. Zolov S. N. Kauffman E. J. Zhang Y. Strunk B. S. Weisman L. S. (2014). Activity-dependent PI(3,5)P2 synthesis controls AMPA receptor trafficking during synaptic depression. *Proc. Natl. Acad. Sci. U.S.A.* 111 E4896–E4905. 10.1073/pnas.1411117111 25355904 PMC4234577

[B124] McPherson P. S. Garcia E. P. Slepnev V. I. David C. Zhang X. Grabs D. (1996). A presynaptic inositol-5-phosphatase. *Nature* 379 353–357. 10.1038/379353a0 8552192

[B125] Mehta Z. B. Pietka G. Lowe M. (2014). The cellular and physiological functions of the Lowe syndrome protein OCRL1. *Traffic* 15 471–487. 10.1111/tra.12160 24499450 PMC4278560

[B126] Ménager C. Arimura N. Fukata Y. Kaibuchi K. (2004). PIP 3 is involved in neuronal polarization and axon formation. *J. Neurochem.* 89 109–118. 10.1046/j.1471-4159.2004.02302.x 15030394

[B127] Menzies F. M. Fleming A. Caricasole A. Bento C. F. Andrews S. P. Ashkenazi A. (2017). Autophagy and neurodegeneration: pathogenic mechanisms and therapeutic opportunities. *Neuron* 93 1015–1034. 10.1016/j.neuron.2017.01.022 28279350

[B128] Mertens J. Wang Q.-W. Kim Y. Yu D. X. Pham S. Yang B. (2015). Differential responses to lithium in hyperexcitable neurons from patients with bipolar disorder. *Nature* 527 95–99. 10.1038/nature15526 26524527 PMC4742055

[B129] Ming G. Song H. Berninger B. Inagaki N. Tessier-Lavigne M. Poo M. (1999). Phospholipase C-gamma and phosphoinositide 3-kinase mediate cytoplasmic signaling in nerve growth cone guidance. *Neuron* 23 139–148. 10.1016/s0896-6273(00)80760-6 10402200

[B130] Miranda A. M. Herman M. Cheng R. Lee J. H. Hussaini S. A. Marquer C. (2018). Excess synaptojanin 1 contributes to place cell dysfunction and memory deficits in the aging hippocampus in three types of Alzheimer’s disease. *Cell Rep.* 23 2967–2975. 10.1016/j.celrep.2018.05.011 29874583 PMC6040810

[B131] Mirzaa G. M. Conway R. L. Gripp K. W. Lerman-sagie T. Siegel D. H. Linda S. (2012). Megalencephaly-capillary malformation (MCAP) and (MPPH) syndromes: two closely related disorders of brain overgrowth and abnormal brain and body morphogenesis. *Am. J. Med. Genet. A* 158 269–291. 10.1002/ajmg.a.34402 22228622

[B132] Morel E. Chamoun Z. Lasiecka Z. M. Chan R. B. Williamson R. L. Vetanovetz C. (2013). Phosphatidylinositol-3-phosphate regulates sorting and processing of amyloid precursor protein through the endosomal system. *Nat. Commun.* 4:2250. 10.1038/ncomms3250 23907271 PMC3905799

[B133] Nahorski M. S. Al-Gazali L. Hertecant J. Owen D. J. Borner G. H. H. Chen Y.-C. (2015). A novel disorder reveals clathrin heavy chain-22 is essential for human pain and touch development. *Brain* 138 2147–2160. 10.1093/brain/awv149 26068709 PMC4511860

[B134] Nakajima J. Okamoto N. Shiraishi J. Nishimura G. Nakashima M. Tsurusaki Y. (2013). Novel FIG4 mutations in Yunis – Varon syndrome. *J. Hum. Genet.* 58 822–824. 10.1038/jhg.2013.104 24088667

[B135] Nakatsu F. Baskin J. M. Chung J. Tanner L. B. Shui G. Lee S. Y. (2012). PtdIns4P synthesis by PI4KIIIα at the plasma membrane and its impact on plasma membrane identity. *J. Cell Biol.* 199 1003–1016. 10.1083/jcb.201206095 23229899 PMC3518224

[B136] Nalls M. A. Pankratz N. Lill C. M. Do C. B. Hernandez D. G. Saad M. (2014). Large-scale meta-analysis of genome-wide association data identifies six new risk loci for Parkinson’s disease. *Nat. Genet.* 46 989–993. 10.1038/ng.3043 25064009 PMC4146673

[B137] Naughtin M. J. Sheffield D. A. Rahman P. Hughes W. E. Gurung R. Stow J. L. (2010). The myotubularin phosphatase MTMR4 regulates sorting from early endosomes. *J. Cell Sci.* 123 3071–3083. 10.1242/jcs.060103 20736309

[B138] Nicholson G. Lenk G. M. Reddel S. W. Grant A. E. Towne C. F. Ferguson C. J. (2011). Distinctive genetic and clinical features of CMT4J: a severe neuropathy caused by mutations in the PI(3,5)P_2_ phosphatase FIG4. *Brain* 134 1959–1971. 10.1093/brain/awr148 21705420 PMC3122378

[B139] Nixon R. A. (2017). Amyloid precursor protein and endosomal-lysosomal dysfunction in Alzheimer’s disease: inseparable partners in a multifactorial disease. *FASEB J.* 31 2729–2743. 10.1096/fj.201700359 28663518 PMC6137496

[B140] Noda T. Matsunaga K. Taguchi-Atarashi N. Yoshimori T. (2010). Regulation of membrane biogenesis in autophagy via PI3P dynamics. *Semin. Cell Dev. Biol.* 21 671–676. 10.1016/j.semcdb.2010.04.002 20403452

[B141] Ohnishi T. Ohba H. Seo K. C. Im J. Sato Y. Iwayama Y. (2007). Spatial expression patterns and biochemical properties distinguish a second myo-inositol monophosphatase IMPA2 from IMPA1. *J. Biol. Chem.* 282 637–646. 10.1074/jbc.M604474200 17068342

[B142] Olgiati S. Rosa A. De Quadri M. Criscuolo C. Breedveld G. J. Picillo M. (2014). PARK20 caused by SYNJ1 homozygous Arg258Gln mutation in a new Italian family. *Neurogenetics* 15 183–188. 10.1007/s10048-014-0406-0 24816432

[B143] Olivos-glander I. M. Janne P. A. Nussbaum R. L. (1995). The oculocerebrorenal syndrome gene product is protein localized to the golgi complex. *Am. J. Hum. Genet.* 57 817–823. 7573041 PMC1801524

[B144] Osborn D. P. S. Pond H. L. Mazaheri N. Dejardin J. Munn C. J. Mushref K. (2017). Mutations in INPP5K cause a form of congenital muscular dystrophy overlapping marinesco-sjögren syndrome and dystroglycanopathy. *Am. J. Hum. Genet.* 100 537–545. 10.1016/j.ajhg.2017.01.019 28190459 PMC5339112

[B145] Othmane K. B. Johnson E. Menold M. Graham F. L. Hamida M. Ben (1999). Identification of a new locus for autosomal recessive Charcot – Marie – tooth disease with focally folded myelin on chromosome 11p15. *Genomics* 62 344–349. 10.1006/geno.1999.6028 10644431

[B146] Pagnamenta A. T. Howard M. F. Wisniewski E. Popitsch N. Knight S. J. L. Keays D. A. (2015). Germline recessive mutations in PI4KA are associated with perisylvian polymicrogyria, cerebellar hypoplasia and arthrogryposis. *Hum. Mol. Genet.* 24 3732–3741. 10.1093/hmg/ddv117 25855803 PMC4459391

[B147] Papadopoulos T. Rhee H. J. Subramanian D. Paraskevopoulou F. Mueller R. Schultz C. (2017). Endosomal phosphatidylinositol 3-phosphate promotes gephyrin clustering and GABAergic neurotransmission at inhibitory postsynapses. *J. Biol. Chem.* 292 1160–1177. 10.1074/jbc.M116.771592 27941024 PMC5270463

[B148] Poduri A. Evrony G. D. Cai X. Elhosary P. C. Beroukhim R. Lehtinen M. K. (2012). Report somatic activation of AKT3 causes hemispheric developmental brain malformations. *Neuron* 74 41–48. 10.1016/j.neuron.2012.03.010 22500628 PMC3460551

[B149] Posor Y. Eichhorn-Gruenig M. Puchkov D. Schöneberg J. Ullrich A. Lampe A. (2013). Spatiotemporal control of endocytosis by phosphatidylinositol-3,4-bisphosphate. *Nature* 499 233–237. 10.1038/nature12360 23823722

[B150] Posor Y. Eichhorn-Grünig M. Haucke V. (2015). Phosphoinositides in endocytosis. *Biochim. Biophys. Acta Mol. Cell Biol. Lipids* 1851 794–804. 10.1016/j.bbalip.2014.09.014 25264171

[B151] Quadri M. Fang M. Picillo M. Olgiati S. Breedveld G. J. Graafland J. (2013). Mutation in the SYNJ1 gene associated with autosomal recessive, early-onset Parkinsonism. *Hum. Mutat.* 34 1208–1215. 10.1002/humu.22373 23804577

[B152] Rahdar M. Inoue T. Meyer T. Zhang J. Vazquez F. Devreotes P. N. (2009). A phosphorylation-dependent intramolecular interaction regulates the membrane association and activity of the tumor suppressor PTEN. *Proc. Natl. Acad. Sci. U.S.A.* 106 480–485. 10.1073/pnas.0811212106 19114656 PMC2626728

[B153] Rameh L. E. Tolias K. F. Duckworth B. C. Cantley L. C. (1997). A new pathway for synthesis of phosphatidylinositol-4,5-bisphosphate. *Nature* 390 192–196. 10.1038/36621 9367159

[B154] Ramel D. Lagarrigue F. Pons V. Mounier J. Dupuis-Coronas S. Chicanne G. (2011). *Shigella flexneri* infection generates the lipid PI5P to alter endocytosis and prevent termination of EGFR signaling. *Sci. Signal.* 4:ra61. 10.1126/scisignal.2001619 21934107

[B155] Rapoport S. I. Primiani C. T. Chen C. T. Ahn K. Ryan V. H. (2015). Coordinated expression of phosphoinositide metabolic genes during development and aging of human dorsolateral prefrontal cortex. *PLoS One* 10:e0132675. 10.1371/journal.pone.0132675 26168237 PMC4500567

[B156] Rhee S. G. (2001). Regulation of phosphoinositide-specific phospholipase C. *Annu. Rev. Biochem.* 70 281–312. 10.1146/annurev.biochem.70.1.281 11395409 PMC4781088

[B157] Rhee S. G. Choi K. D. (1992). Regulation of inositol phospholipid-specific phospholipase C isozymes. *J. Biol. Chem.* 267 12393–12396. 1319994

[B158] Ripke S. Neale B. M. Corvin A. Walters J. T. R. Farh K.-H. Holmans P. A. (2014). Biological insights from 108 schizophrenia-associated genetic loci. *Nature* 511 421–427. 10.1038/nature13595 25056061 PMC4112379

[B159] Robinson F. L. Dixon J. E. (2005). The phosphoinositide-3-phosphatase MTMR2 associates with MTMR13, a membrane-associated pseudophosphatase also mutated in type 4B Charcot-Marie-tooth disease. *J. Biol. Chem.* 280 31699–31707. 10.1074/jbc.M505159200 15998640

[B160] Ruderfer D. M. Fanous A. H. Ripke S. Mcquillin A. Amdur R. L. Working S. (2014). Polygenic dissection of diagnosis and clinical dimensions of bipolar disorder and Schizophrenia. *Mol. Psychiatry* 19 1017–1024. 10.1038/mp.2013.138 24280982 PMC4033708

[B161] Russo F. B. Cugola F. R. Fernandes I. R. Pignatari G. C. Beltrão-Braga P. C. B. (2015). Induced pluripotent stem cells for modeling neurological disorders. *World J. Transplant.* 5 209–221. 10.5500/wjt.v5.i4.209 26722648 PMC4689931

[B162] Saiardi A. Mudge A. W. (2018). Lithium and fluoxetine regulate the rate of phosphoinositide synthesis in neurons: a new view of their mechanisms of action in bipolar disorder. *Transl. Psychiatry* 8:175. 10.1038/s41398-018-0235-2 30171184 PMC6119186

[B163] Sanchez-Juan P. Bishop M. T. Aulchenko Y. S. Brandel J.-P. Rivadeneira F. Struchalin M. (2012). Genome-wide study links MTMR7 gene to variant Creutzfeldt-Jakob risk. *Neurobiol. Aging* 33:1487.e21-.e28. 10.1016/j.neurobiolaging.2011.10.011 22137330

[B164] Sansal I. Sellers W. R. (2014). The biology and clinical relevance of the PTEN tumor suppressor pathway. *J. Clin. Oncol.* 22 2954–2963. 10.1200/JCO.2004.02.141 15254063

[B165] Sarkes D. Rameh L. E. (2010). A novel HPLC-based approach makes possible the spatial characterization of cellular PtdIns5P and other phosphoinositides. *Biochem. J.* 428 375–384. 10.1042/BJ20100129 20370717 PMC2944655

[B166] Sasaki J. Kofuji S. Itoh R. Momiyama T. Takayama K. Murakami H. (2010). The PtdIns(3,4)P(2) phosphatase INPP4A is a suppressor of excitotoxic neuronal death. *Nature* 465 497–501. 10.1038/nature09023 20463662

[B167] Sasaki T. Takasuga S. Sasaki J. Kofuji S. Eguchi S. Yamazaki M. (2009). Mammalian phosphoinositide kinases and phosphatases. *Prog. Lipid Res.* 48 307–343. 10.1016/j.plipres.2009.06.001 19580826

[B168] Schaletzky J. Dove S. K. Short B. Lorenzo O. Clague M. J. Barr F. A. (2003). Phosphatidylinositol-5-phosphate activation and conserved substrate specificity of the myotubularin phosphatidylinositol 3-phosphatases. *Curr. Biol.* 13 504–509. 10.1016/S0960-9822(03)00132-5 12646134

[B169] Schu P. V. Takegawa K. Fry M. J. Stack J. H. Waterfield M. D. Emr S. D. (1993). Phosphatidylinositol 3-kinase encoded by yeast VPS34 gene essential for protein sorting. *Science* 260 88–91. 10.1126/science.8385367 8385367

[B170] Schwab S. G. Knapp M. Sklar P. Eckstein G. N. Sewekow C. Borrmann-Hassenbach M. (2006). Evidence for association of DNA sequence variants in the phosphatidylinositol-4-phosphate 5-kinase IIalpha gene (PIP5K2A) with schizophrenia. *Mol. Psychiatry* 11 837–846. 10.1038/sj.mp.4001864 16801950

[B171] Seebohm G. Neumann S. Theiss C. Novkovic T. Hill E. V. Tavaré J. M. (2012). Identification of a novel signaling pathway and its relevance for GluA1 recycling. *PLoS One* 7:e33889. 10.1371/journal.pone.0033889 22470488 PMC3309939

[B172] Sherva R. Wang Q. Kranzler H. Zhao H. Koesterer R. Herman A. (2016). Genome-wide association study of cannabis dependence severity, novel risk variants, and shared genetic risks. *JAMA Psychiatry* 73 472–480. 10.1001/jamapsychiatry.2016.0036 27028160 PMC4974817

[B173] Shetty M. Nimmy R. Shubhi S. Mullapudi N. Hegde S. (2017). A homozygous missense variant in INPP5E associated with joubert syndrome and related disorders. *Mol. Syndromol.* 8 313–317. 10.1159/000479673 29230161 PMC5701266

[B174] Shi Y. Inoue H. Wu J. C. Yamanaka S. (2017). Induced pluripotent stem cell technology: a decade of progress. *Nat. Rev. Drug Discov.* 16 115–130. 10.1038/nrd.2016.245 27980341 PMC6416143

[B175] Shisheva A. Sbrissa D. Ikonomov O. (2015). Plentiful PtdIns5P from scanty PtdIns(3,5)P 2 or from ample PtdIns? PIKfyve-dependent models: evidence and speculation (response to: DOI 10.1002/bies.201300012). *BioEssays* 37 267–277. 10.1002/bies.201400129 25404370 PMC4636131

[B176] Simon G. E. (2003). Social and economic burden of mood disorders. *Biol. Psychiatry* 54 208–215. 10.1016/S0006-3223(03)00420-7 12893097

[B177] Simons J. P. Al-Shawi R. Minogue S. Waugh M. G. Wiedemann C. Evangelou S. (2009). Loss of phosphatidylinositol 4-kinase 2α activity causes late onset degeneration of spinal cord axons. *Proc. Natl. Acad. Sci. U.S.A.* 106 11535–11539. 10.1073/pnas.0903011106 19581584 PMC2710652

[B178] Simonsen A. Wurmser A. E. Emr S. D. Stenmark H. (2001). The role of phosphoinositides in membrane transport. *Curr. Opin. Cell Biol.* 13 485–492. 10.1016/S0955-0674(00)00240-4 11454456

[B179] Soares J. C. Mallinger A. G. (1995). Abnormal phosphatidylinositol (PI)-signalling in bipolar disorde. *Biol. Psychiatry* 3223 1995–1996. 10.1016/0006-3223(95)00527-7 8679795

[B180] Soutar M. P. M. Kempthorne L. Miyakawa S. Annuario E. Melandri D. Harley J. (2018). AKT signalling selectively regulates PINK1 mitophagy in SHSY5Y cells and human iPSC-derived neurons. *Sci. Rep.* 8:8855. 10.1038/s41598-018-26949-6 29891871 PMC5995958

[B181] Splinter K. Adams D. R. Bacino C. A. Bellen H. J. Bernstein J. A. Cheatle-Jarvela A. M. (2018). Effect of genetic diagnosis on patients with previously undiagnosed disease. *N. Engl. J. Med.* 379 2131–2139. 10.1056/NEJMoa1714458 30304647 PMC6481166

[B182] Stefan C. J. Manford A. G. Baird D. Yamada-Hanff J. Mao Y. Emr S. D. (2011). Osh proteins regulate phosphoinositide metabolism at ER-plasma membrane contact sites. *Cell* 144 389–401. 10.1016/j.cell.2010.12.034 21295699

[B183] Thiselton D. L. Maher B. S. Webb B. T. Bigdeli T. B. O’Neill F. A. Walsh D. (2009). Association analysis of the *PIP4K2A* gene on chromosome 10p12 and Schizophrenia in the Irish study of high density schizophrenia families (ISHDSF) and the Irish case-control study of schizophrenia (ICCSS). *Am. J. Med. Genet. Part B Neuropsychiatr. Genet.* 153B 323–331. 10.1002/ajmg.b.30982 19475563 PMC4011176

[B184] Tighe S. K. Mahon P. B. Potash J. B. (2011). Predictors of lithium response in bipolar disorder. *Ther. Adv. Chronic Dis.* 2 209–226. 10.1177/2040622311399173 23251751 PMC3513882

[B185] Tohyama J. Nakashima M. Nabatame S. Gaik-Siew C. Miyata R. Rener-Primec Z. (2015). SPTAN1 encephalopathy: distinct phenotypes and genotypes. *J. Hum. Genet.* 60 167–173. 10.1038/jhg.2015.5 25631096

[B186] Travaglini L. Brancati F. Silhavy J. Iannicelli M. Nickerson E. Elkhartoufi N. (2013). Phenotypic spectrum and prevalence of INPP5E mutations in Joubert syndrome and related disorders. *Eur. J. Hum. Genet.* 21 1074–1078. 10.1038/ejhg.2012.305 23386033 PMC3778343

[B187] Tsuruta F. Green E. M. Rousset M. Dolmetsch R. E. (2009). PIKfyve regulates CaV1.2 degradation and prevents excitotoxic cell death. *J. Cell Biol.* 187 279–294. 10.1083/jcb.200903028 19841139 PMC2768838

[B188] Vaccari I. Dina G. Tronchère H. Kaufman E. Chicanne G. Cerri F. (2011). Genetic interaction between MTMR2 and FIG4 phospholipid phosphatases involved in Charcot-Marie-Tooth neuropathies. *PLoS Genet.* 7:e1002319. 10.1371/journal.pgen.1002319 22028665 PMC3197679

[B189] van den Bout I. Divecha N. (2009). PIP5K-driven PtdIns(4,5)P2 synthesis: regulation and cellular functions. *J. Cell Sci.* 122 3837–3850. 10.1242/jcs.056127 19889969

[B190] van Diepen M. T. Eickholt B. J. (2008). Function of PTEN during the formation and maintenance of neuronal circuits in the brain. *Dev. Neurosci.* 30 59–64. 10.1159/000109852 18075255

[B191] Vanhaesebroeck B. Guillermet-Guibert J. Graupera M. Bilanges B. (2010). The emerging mechanisms of isoform-specific PI3K signalling. *Nat. Rev. Mol. Cell Biol.* 11 329–341. 10.1038/nrm2882 20379207

[B192] Varga E. A. Pastore M. Prior T. Herman G. E. (2009). The prevalence of PTEN mutations in a clinical pediatric cohort with autism spectrum disorders, developmental delay, and macrocephaly. *Genet. Med.* 11 111–117. 10.1097/GIM.0b013e31818fd762 19265751

[B193] Vaucher J. Keating B. J. Lasserre A. M. Gan W. Lyall D. M. Ward J. (2017). Cannabis use and risk of schizophrenia: a Mendelian randomization study. *Nat. Publ. Gr.* 23 1287–1292. 10.1038/mp.2016.252 28115737 PMC5984096

[B194] Vehlow A. Soong D. Vizcay-Barrena G. Bodo C. Law A.-L. Perera U. (2013). Endophilin, Lamellipodin, and Mena cooperate to regulate F-actin-dependent EGF-receptor endocytosis. *EMBO J.* 32 2722–2734. 10.1038/emboj.2013.212 24076656 PMC3801443

[B195] Voronov S. V. Frere S. G. Giovedi S. Pollina E. A. Borel C. Zhang H. (2008). Synaptojanin 1-linked phosphoinositide dyshomeostasis and cognitive deficits in mouse models of Down’s syndrome. *Proc. Natl. Acad. Sci. U.S.A.* 105 9415–9420. 10.1073/pnas.0803756105 18591654 PMC2453748

[B196] Walch-Solimena C. Novick P. (1999). The yeast phosphatidylinositol-4-OH kinase pik1 regulates secretion at the Golgi. *Nat. Cell Biol.* 1 523–525. 10.1038/70319 10587649

[B197] Walker D. M. Urbé S. Dove S. K. Tenza D. Raposo G. Clague M. J. (2001). Characterization of MTMR3. an inositol lipid 3-phosphatase with novel substrate specificity. *Curr. Biol.* 11 1600–1605. 10.1016/S0960-9822(01)00501-2 11676921

[B198] Wang L. Budolfson K. Wang F. (2011). Pik3c3 deletion in pyramidal neurons results in loss of synapses, extensive gliosis and progressive neurodegeneration. *Neuroscience* 172 427–442. 10.1016/j.neuroscience.2010.10.035 20955765 PMC3010427

[B199] Wang Y. J. Wang J. Sun H. Q. Martinez M. Sun Y. X. Macia E. (2003). Phosphatidylinositol 4 phosphate regulates targeting of clathrin adaptor AP-1 complexes to the Golgi. *Cell* 114 299–310. 10.1016/s0092-8674(03)00603-2 12914695

[B200] Wen Z. Christian K. M. Song H. Ming G. (2016). Modeling psychiatric disorders with patient-derived iPSCs. *Curr. Opin. Neurobiol.* 36 118–127. 10.1016/j.conb.2015.11.003 26705693 PMC4738077

[B201] Wenk M. R. Pellegrini L. Klenchin V. A. Di Paolo G. Chang S. Daniell L. (2001). PIP kinase Igamma is the major PI(4,5)P(2) synthesizing enzyme at the synapse. *Neuron* 32 79–88. 10.1016/s0896-6273(01)00456-1 11604140

[B202] Wiessner M. Roos A. Munn C. J. Viswanathan R. Whyte T. Cox D. (2017). Mutations in INPP5K, encoding a phosphoinositide 5-phosphatase, cause congenital muscular dystrophy with cataracts and mild cognitive impairment. *Am. J. Hum. Genet.* 100 523–536. 10.1016/j.ajhg.2017.01.024 28190456 PMC5339217

[B203] Wu C. Y. Lin M. W. Wu D. C. Huang Y. B. Huang H. T. Chen C. L. (2014). The role of phosphoinositide-regulated actin reorganization in chemotaxis and cell migration. *Br. J. Pharmacol.* 171 5541–5554. 10.1111/bph.12777 25420930 PMC4290701

[B204] Xie T. Deng L. Mei P. Zhou Y. Wang B. Zhang J. (2014). A genome-wide association study combining pathway analysis for typical sporadic amyotrophic lateral sclerosis in Chinese Han populations. *Neurobiol. Aging* 35:1778.e9-e23. 10.1016/j.neurobiolaging.2014.01.014 24529757

[B205] Yoshinaga S. Ohkubo T. Sasaki S. Nuriya M. Ogawa Y. Yasui M. (2012). A phosphatidylinositol lipids system, lamellipodin, and Ena/VASP regulate dynamic morphology of multipolar migrating cells in the developing cerebral cortex. *J. Neurosci.* 32 11643–11656. 10.1523/JNEUROSCI.0738-12.2012 22915108 PMC6703763

[B206] Zewe J. P. Wills R. C. Sangappa S. Goulden B. D. Hammond G. R. (2018). SAC1 degrades its lipid substrate PtdIns4P in the endoplasmic reticulum to maintain a steep chemical gradient with donor membranes. *eLife* 7:e35588. 10.7554/eLife.35588 29461204 PMC5829913

[B207] Zhang S. Duan L. He S. Zhuang G. Yu X. (2017). Phosphatidylinositol 3, 4-bisphosphate regulates neurite initiation and dendrite morphogenesis via actin aggregation. *Nat. Publ. Gr.* 27 253–273. 10.1038/cr.2017.13 28106075 PMC5339852

[B208] Zhang X. Wang W.-A. Jiang L.-X. Liu H.-Y. Zhang B.-Z. Lim N. (2017). Downregulation of RBO-PI4KIIIα facilitates Aβ 42 secretion and ameliorates neural deficits in Aβ 42 -expressing *Drosophila*. *J. Neurosci.* 37 4928–4941. 10.1523/JNEUROSCI.3567-16.2017 28424219 PMC6596482

[B209] Zhang X. Clement Y. Sahenk Z. Shy M. E. Meisler M. H. Li J. (2008). Mutation of FIG4 causes a rapidly progressive, asymmetric neuronal degeneration. *Brain* 131 1990–2001. 10.1093/brain/awn114 18556664 PMC2724900

[B210] Zhang Y. Sloan S. A. Clarke L. E. Caneda C. Plaza C. A. Blumenthal P. D. (2016). Purification and characterization of progenitor and mature human astrocytes reveals transcriptional and functional differences with mouse. *Neuron* 89 37–53. 10.1016/j.neuron.2015.11.013 26687838 PMC4707064

[B211] Zheng L. Conner S. D. (2018). PI5P4Kγ functions in DTX1-mediated Notch signaling. *Proc. Natl. Acad. Sci. U.S.A.* 115 E1983–E1990. 10.1073/pnas.1712142115 29440432 PMC5834675

[B212] Zhou X. Wang L. Hasegawa H. Amin P. Han B. Kaneko S. (2010). Deletion of PIK3C3/Vps34 in sensory neurons causes rapid neurodegeneration by disrupting the endosomal but not the autophagic pathway. *Proc. Natl. Acad. Sci. U.S.A.* 107 9424–9429. 10.1073/pnas.0914725107 20439739 PMC2889054

[B213] Zolov S. N. Bridges D. Zhang Y. Lee W. Riehle E. Verma R. (2012). In vivo, Pikfyve generates PI(3,5)P2, which serves as both a signaling lipid and the major precursor for PI5P. *Proc. Natl. Acad. Sci. U.S.A.* 109 17472–17477. 10.1073/pnas.1203106109 23047693 PMC3491506

